# Microbial Diversity, Community Turnover, and Putative Functions in Submarine Canyon Sediments under the Action of Sedimentary Geology

**DOI:** 10.1128/spectrum.04210-22

**Published:** 2023-02-21

**Authors:** Hualin Liu, Xueyu Cai, Kunwen Luo, Sihan Chen, Ming Su, Jianguo Lu

**Affiliations:** a School of Marine Sciences, Sun Yat-sen University, Zhuhai, China; b Southern Marine Science and Engineering Guangdong Laboratory (Zhuhai), Zhuhai, China; c Guangdong Provincial Key Laboratory of Marine Resources and Coastal Engineering, Guangzhou Guangdong, China; d Pearl River Estuary Marine Ecosystem Research Station, Ministry of Education, Zhuhai, China; Nanjing Institute of Geography and Limnology Chinese Academy of Sciences

**Keywords:** biogeochemical cycle, ecological processes, South China Sea, turbidity currents

## Abstract

Sampling challenges in deep-sea ecosystems lead to a lack of knowledge about the distribution of microbes in different submarine canyons. To study microbial diversity and community turnover under different ecological processes, we performed 16S/18S rRNA gene amplicon sequencing for sediment samples from a submarine canyon in the South China Sea. Bacteria, archaea, and eukaryotes made up 57.94% (62 phyla), 41.04% (12 phyla), and 1.02% (4 phyla) of the sequences, respectively. Thaumarchaeota, Planctomycetota, *Proteobacteria*, Nanoarchaeota, and *Patescibacteria* are the five most abundant phyla. Heterogeneous community composition was mainly observed in vertical profiles rather than horizontal geographic locations, and microbial diversity in the surface layer was much lower than that in deep layers. According to the null model tests, homogeneous selection dominated community assembly within each sediment layer, whereas heterogeneous selection and dispersal limitation dominated community assembly between distant layers. Different sedimentation processes of sediments, i.e., rapid deposition caused by turbidity currents or slow sedimentation, seem to be primarily responsible for these vertical variations. Finally, functional annotation through shotgun-metagenomic sequencing found that glycosyl transferases and glycoside hydrolases are the most abundant carbohydrate-active enzyme categories. The most likely expressed sulfur cycling pathways include assimilatory sulfate reduction, the link between inorganic and organic sulfur transformation, and organic sulfur transformation, while the potentially activated methane cycling pathways include aceticlastic methanogenesis and aerobic and anaerobic oxidation of methane. Overall, our study revealed high levels of microbial diversity and putative functions in canyon sediments and the important influence of sedimentary geology on microbial community turnover between vertical sediment layers.

**IMPORTANCE** Deep-sea microbes have received growing attention due to their contribution to biogeochemical cycles and climate change. However, related research lags due to the difficulty of collecting samples. Based on our previous study, which revealed the formation of sediments under the dual action of turbidity currents and seafloor obstacles in a submarine canyon in the South China Sea, this interdisciplinary research provides new insights into how sedimentary geology influences microbial community assembly in sediments. We proposed some uncommon or new findings, including the following: (i) microbial diversity was much lower on the surface than in deeper layers (ii) archaea and bacteria dominated the surface and deep layers, respectively; (iii) sedimentary geology played key roles in vertical community turnover; and (iv) the microbes have great potential to catalyze sulfur, carbon, and methane cycling. This study may lead to extensive discussion of the assembly and function of deep-sea microbial communities in the context of geology.

## INTRODUCTION

Deep-sea ecosystems represent the largest biome of the global biosphere and host yet undiscovered biodiversity (1). It is one of the most important extreme environments, characterized by high hydrostatic pressure, high salinity, low temperature, darkness, and oligotrophic conditions (2, 3). Deep-sea microbes make up 90% of the Earth’s microbial population, and about two-thirds of the planet’s microbes inhabit the subseafloor biosphere (4, 5). The prokaryotic cells in the first few meters of the global subseafloor is estimated to be 2.9 × 10^29^ (6) and about half of the oceanic microbial cells are found in sediments (7).

The submarine canyon is one of the most active deep-sea habitats, acting as a conduit for material transport from the coast or shelf to the deep sea (8, 9). It is also a key issue in studying the impact of shelf/deep-sea particulate pollutant exchange on marine ecosystems and global geochemical cycling (10). Bacteria dominate the biomass in canyon sediments and play critical roles in material cycling and energy transfer (11, 12). Compared to the open continental slope, the microbial assemblages and their functional profiles in the canyon showed higher heterogeneity and temporal variability (13), and higher prokaryotic abundance was also observed in the canyon than in the open slope at comparable depths (14). Although some factors have been found to influence the diversity and abundance of benthic deep-sea microbes (15, 16), the drivers that control the diversity and distribution of microorganisms in submarine canyons remain largely unexplored due to the difficulty of accessing the deep seafloor and determining stochastic physical phenomena (14, 17, 18).

Benthic ecosystems are sensitive to environmental changes caused by physical forcings, such as turbidity currents, open sea convection, and dense shelf water cascading events (15, 19). Recently, sediment push cores in the bending area of a canyon located on the continental slope of the northern South China Sea (SCS) were collected by the manned submersible vehicle *Deep-Sea Warrior*. Geomorphological research (20) has revealed the effects of seamount and turbidity currents on the formation of the bending of the canyon and sediment waves, providing us with the opportunity to study how sedimentary geology influences microbial community assembly. In short, much evidence suggests that the sedimentation of deep sediments was influenced by turbidity currents and Wanhu Seamount, while the upper sediments were mainly formed by slow sedimentation without the influence of recent turbidity currents. This study performed 16S/18S rRNA gene sequencing and shotgun-metagenomic sequencing using sediment samples from the same bending area to provide a mechanistic understanding of microbial diversity, community assembly, and putative functions in the submarine canyon system influenced by sedimentary processes and the seamount. Our study may lead to extensive discussion of the assembly and function of deep-sea microbial communities in the context of geology.

## RESULTS

### Microbial diversity.

**FIG 1:**
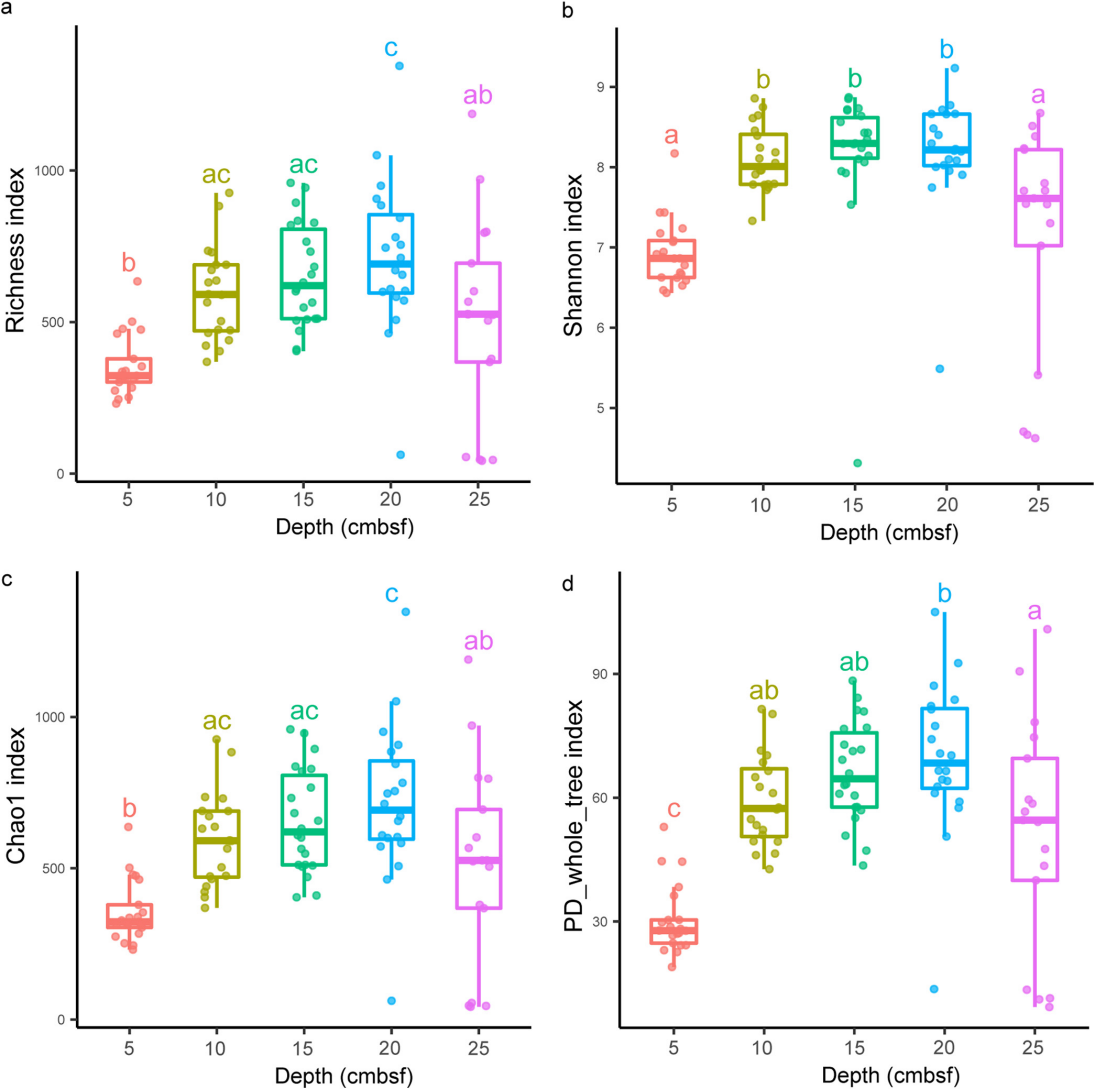
(a to d) Comparison of diversity between different layers of the sediment cores: (a) richness, (b) Shannon index, (c) Chao 1 index, and (d) phylogenetic diversity. Significant differences between groups are indicated by lowercase letters (adjusted *P* < 0.05, analysis of variance [ANOVA], Tukey HSD test). The number on the horizontal axis represents the depth interval; for example, 5 means 0 to 5 cmbsf, 10 means 5 to 10 cmbsf, and so on.

A total of 23,786 amplicon sequence variants (ASVs) were found, and 22,874 remained after the removal of 912 that were unassigned to bacteria, archaea, and eukaryotes. Alpha diversity was compared between vertical sediment layers and between horizontal push cores (Fig. 1 and Fig. S1 in the supplemental material). The diversity between push cores showed no significant difference (the Tukey honestly significant difference [HSD] test), while the top 5 centimeters (0 to 5 cm below seafloor [cmbsf]) had lower diversity than the other layers. With increasing depth, microbial diversity gradually increased, peaking at 15 to 20 cmbsf, and then decreased. There were 21,645 ASVs left after removal of singletons, doubletons, and unassigned ASVs. Subsamples B17, D21, D24, D25, and D26, which contain very few ASVs, were excluded from the following analysis. Finally, a total of 57.94% bacteria (62 phyla), 41.04% archaea (12 phyla), and 1.02% eukaryotes (4 phyla) were recovered.

**FIG 2:**
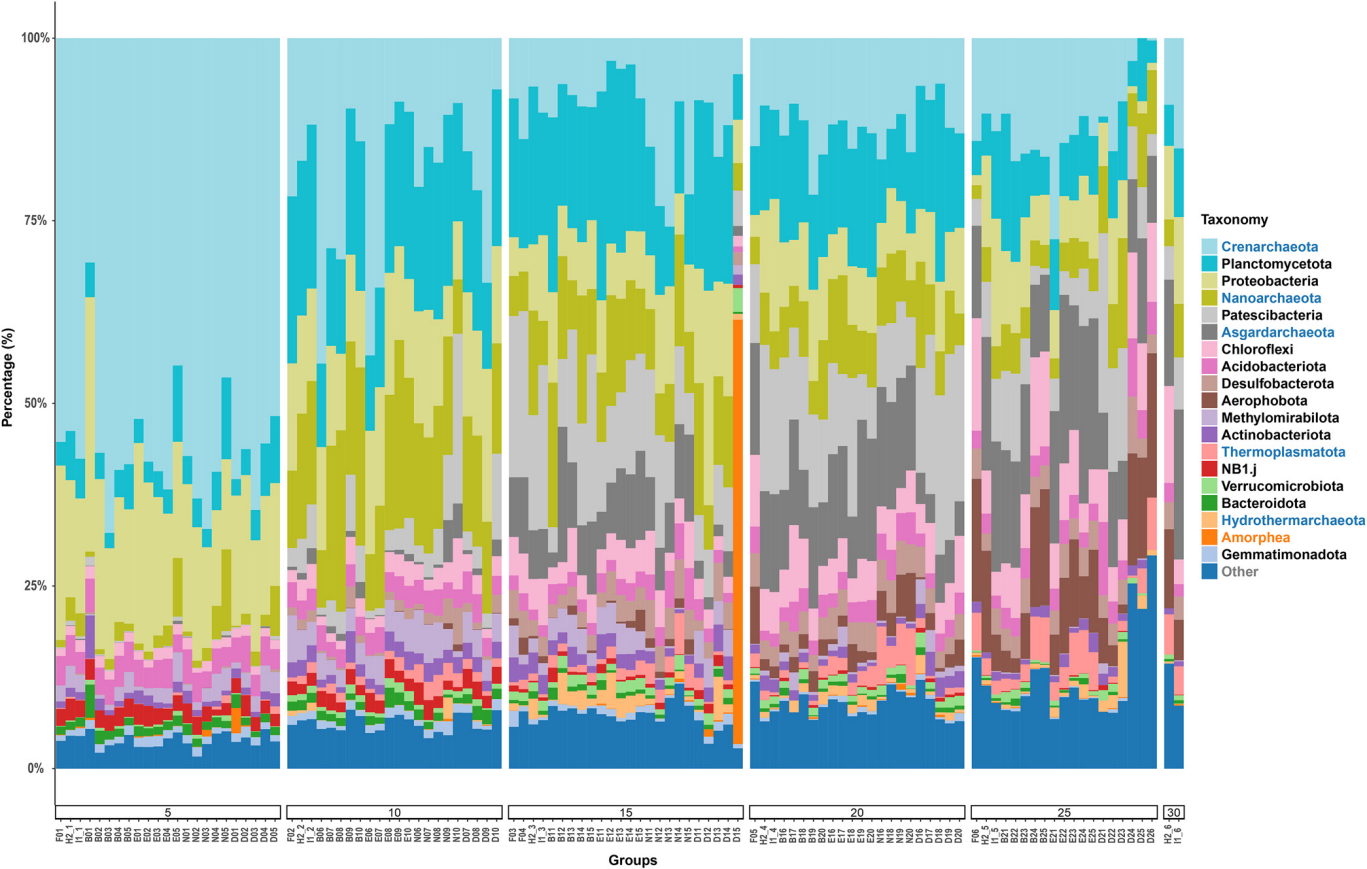
Relative abundances of sequences at the phylum level. The font color of the phyla on the right represents the three kingdoms: archaea are colored blue, bacteria are colored black, and eukaryotes are colored orange. Samples were grouped by sediment depth (cmbsf). The Crenarchaeota here consist mainly of mesophilic Crenarchaeota (now called Thaumarchaeota, but still called Crenarchaeota in the SILVA v138.1 database).

The 19 most abundant phyla, accounting for 92.66% of all sequences, are shown in Fig. 2 and Fig. S2. Archaea and bacteria show similar overall relative abundances in the subseafloor sediments. The relative abundance of archaea was higher than that of bacteria only in the surface layer (0 to 5 cmbsf) (Fig. S3). This trend is embodied in the uneven distribution of several abundant taxa in this study. Crenarchaeota (consisting of 82.49% Nitrososphaeria, 16.76% Bathyarchaeia, 0.04% Thermoprotei, and 0.72% unclassified ASVs) contributed more than 50% of the clones in the surface 0 to 5 cmbsf (Fig. 2). The class Nitrososphaeria, belonging to the phylum Crenarchaeota in the Silva v138.1 database (21), has now been classified into phylum Thaumarchaeota, which was originally thought to be mesophilic Crenarchaeota (also known as MG-1) and was later defined as the third archaeal phylum (22, 23). The most abundant class, Nitrososphaeria, which consists mainly of the family Nitrosopumilaceae, has the highest relative abundance (>50% of all clones) in the surface sediments (Fig. S4). Its relative abundance decreases gradually with increasing sediment depth. The classes *Dehalococcoidia* and *Anaerolineae*, accounting for 97.23% of the *Chloroflexi*, showed increasing abundance with increasing depth. The relative abundance of *Planctomycetota* peaked in the intermediate layers before declining in the deep layers. The relative abundance of *Desulfobacterota* was also positively correlated with depth. We also studied the diversity of eukaryotic microbes, considering their influence on the breakdown of organic carbon (24). In general, the relative abundance of Eukaryota is very low (1.02%), although an abnormally high abundance of Amorphea occurred in sample D15 (14 to 15 cmbsf) (Fig. 2). It is worth noting that the sequences of Eukaryota were present from the surface to 30 cmbsf, and their relative abundance in each layer was also similar.

### Community turnover patterns.

**FIG 3:**
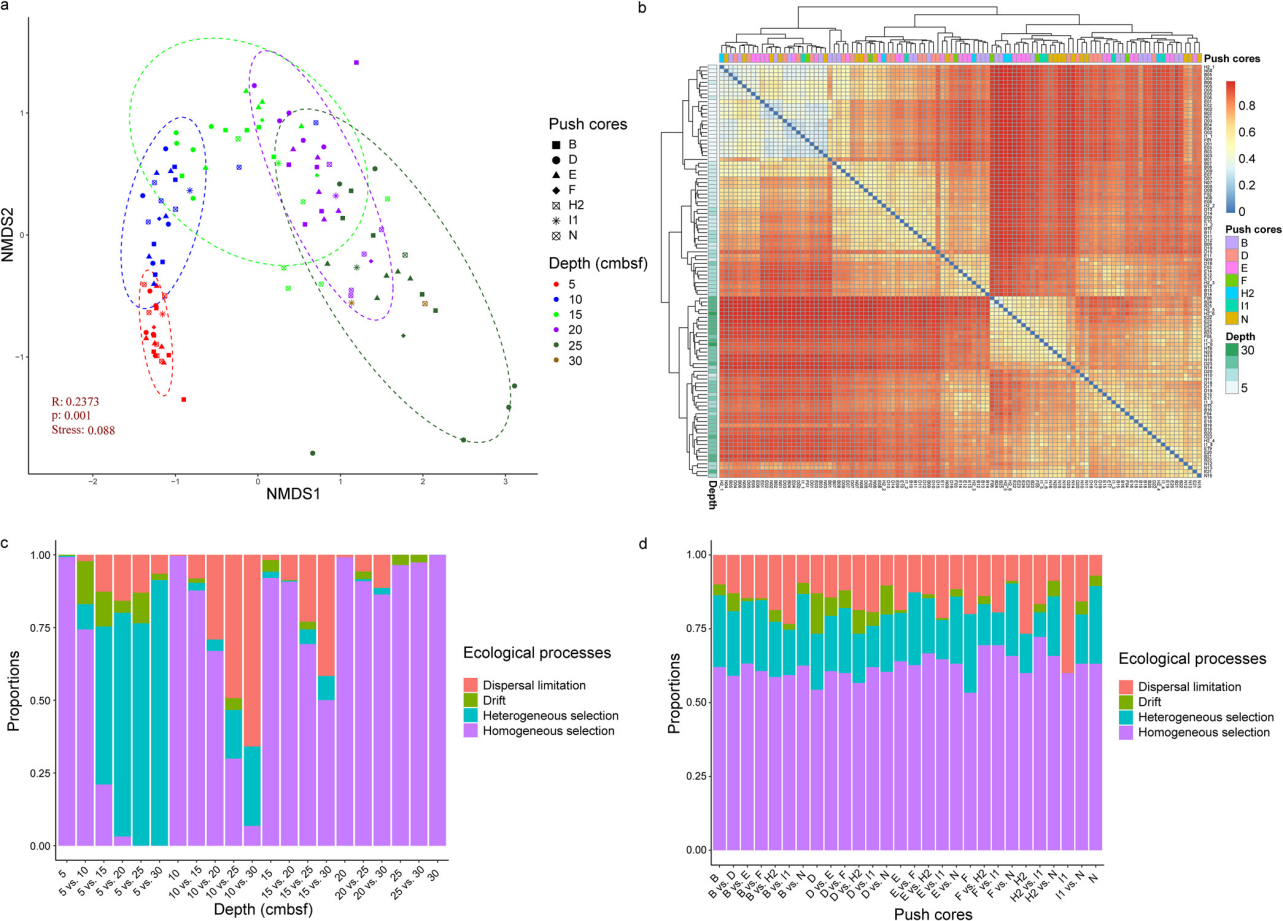
Community assembly patterns and the influencing factors. (a and b) The nonmetric multidimensional scaling (NMDS) diagram (a) and heatmap (b) based on Bray-Curtis dissimilarity show that sample clustering is strongly depth dependent and not geographic location dependent. (c and d) The community assembly framework analysis, which focused on sediment layers (c) and push cores (d), brings insight into the contributions of each ecological process to the community assembly.

Nonmetric multidimensional scaling (NMDS) analysis based on Bray-Curtis dissimilarity was performed to reveal the community turnover pattern. Samples are clustered by depth rather than push cores (Fig. 3a and b). In detail, subsamples from the same depth or adjacent layers, even from different push cores, were clustered together, while subsamples between the top layers (0 to 10 cmbsf) and the bottom layers (15 to 30 cmbsf) were difficult to get together. The middle layers (10 to 15 cmbsf) seemed like transition layers, and many subsamples from the bottom layers were mixed on the plot (Fig. 3a). We performed the null model test to estimate the contributions of each ecological process to community assembly within and between sediment layers and push cores. Significant fluctuations were observed between the sediment layers (Fig. 3c). Homogeneous selection almost completely controlled community assembly within sediment layers and composed more than 50% of the total processes in or between the push cores (Fig. 3c and d). With increasing depth interval, the proportion of homogeneous selection gradually decreased and was replaced by an increase in heterogeneous selection and dispersal limitation. Community turnover between the surface layer (0 to 5 cmbsf) and the deeper layers (10 to 30 cmbsf) was mainly influenced by heterogeneous selection, while dispersal limitation played an increasing role in community turnover between 5 to 10 cmbsf and 15 to 30 cmbsf. Similar patterns were observed within and between the push cores; that is, homogeneous selection was the dominant process, followed by heterogeneous selection, dispersal limitation, and drift. An exception was that the community assembly in push core I1 was fully influenced by homogeneous selection and dispersal limitation.

### Carbon, nitrogen, sulfur, and methane cycling in sediments.

**FIG 4:**
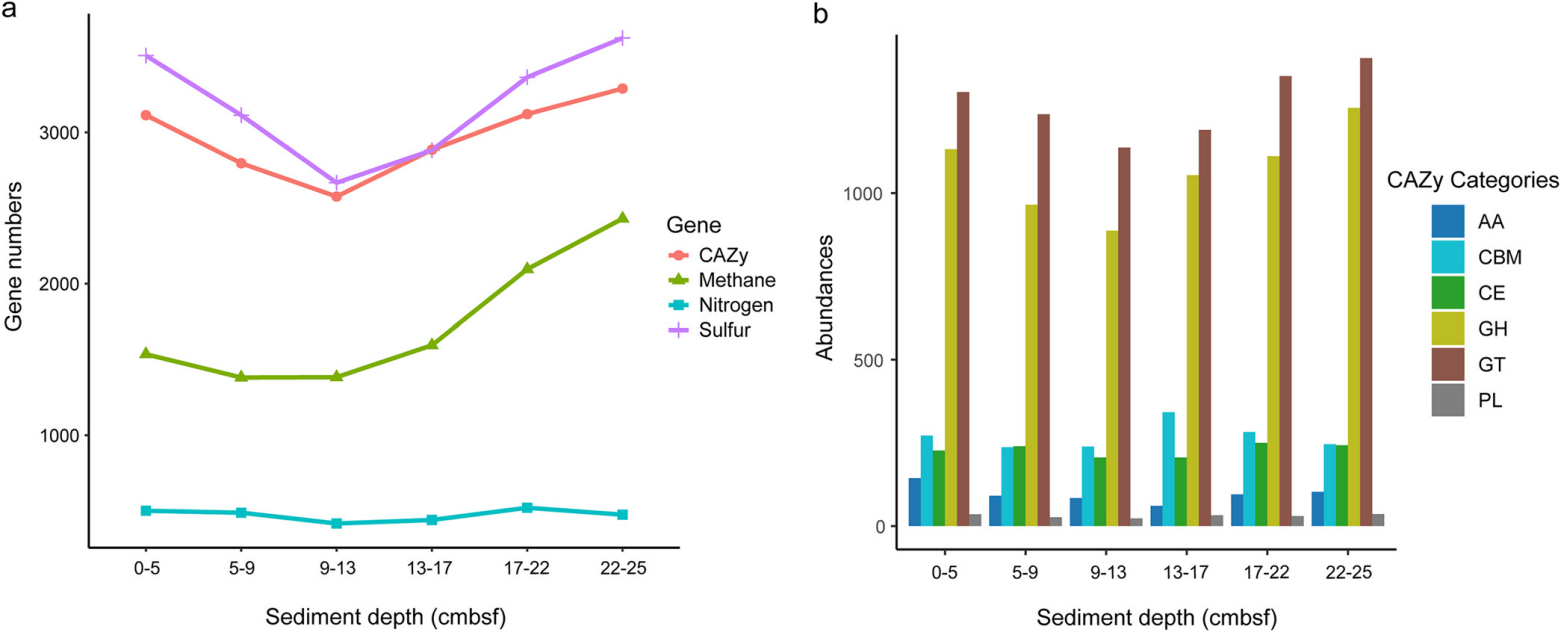
Putative functions encoded by microorganisms. (a) Variation in the number of genes related to the metabolism of various substances. (b) Carbohydrate-Active Enzyme categories in sediment layers of core F predicted by metagenomic sequencing. The total number of sequences was normalized to 42,996 per sample for analysis. AA, auxiliary activities; CBM, carbohydrate-binding modules; CE, carbohydrate esterase; GH, glycoside hydrolases; GT, glycosyl transferases; PL, polysaccharide lyases.

According to the alpha and beta diversity analysis, microbial community turnover mainly occurred in vertical profiles rather than between horizontal push cores. Thus, shotgun-metagenomic sequencing was performed on six subsamples of push core F as a representative to identify the potential for microbial participation in carbon, nitrogen, sulfur, and methane cycling. The assembly statistics of the metagenomes are shown in Table S2. Overall, the number of genes involved in sulfur cycling was the largest, followed by carbohydrate-active enzymes and genes involved in methane cycling, while the number of genes involved in nitrogen cycling was very small (see File S1). With increasing sediment depth, the number of genes for the cycling of sulfur, carbon, and methane first decreased and then increased, and methane cycling genes were more abundant at the bottom than in the surface layer, while the number of genes related to nitrogen cycling remained almost unchanged (Fig. 4a and Table S3).

### Organic carbon metabolism.

The Carbohydrate-Active enZYmes (CAZy) database (25) was employed for functional gene mining to estimate the sedimentary microbes’ complex carbohydrate degradation and metabolism activities. Glycosyl transferases (GT; formation of glycosidic bonds; 60 families) and glycoside hydrolases (GH; hydrolysis and/or rearrangement of glycosidic bonds; 72 families and 177 subfamilies) are the most abundant CAZy categories, followed by carbohydrate-binding modules (CBM; adhesion to carbohydrates; 40 families), carbohydrate esterase (CE; hydrolysis of carbohydrate esters; 12 families), auxiliary activities (AA; redox enzymes acting in conjunction with CAZymes; 11 families and 16 subfamilies), and polysaccharide lyases (PL; nonhydrolytic cleavage of glycosidic bonds; 23 families and 39 subfamilies) (Fig. 4b and Table S4). GT4, CE8, GH23, and GT2 are the four most abundant enzymes (Table S5 and Fig. S5).

### Sulfur cycling.

**FIG 5:**
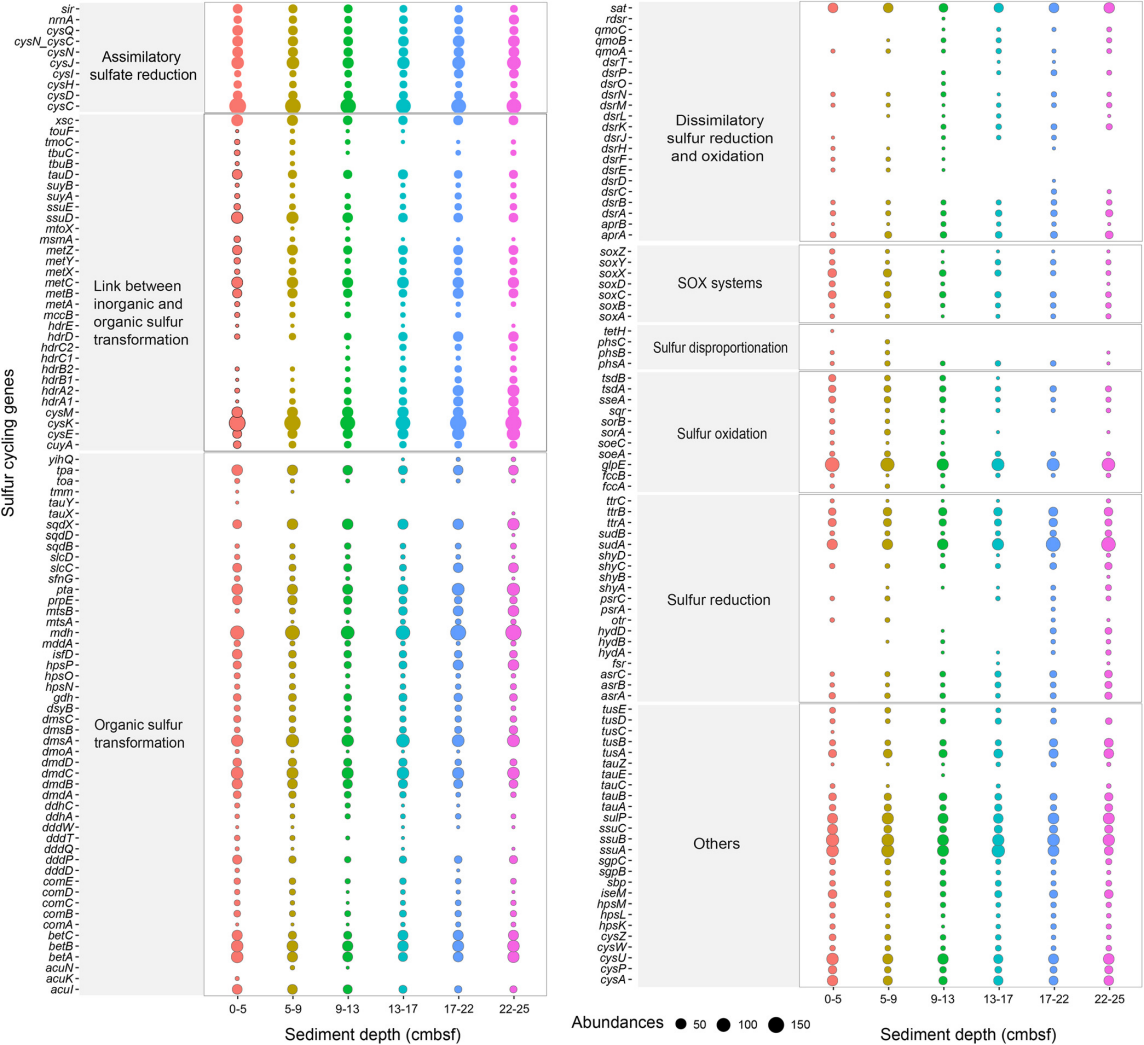
Sulfur cycling genes in sediment layers of push core F predicted by metagenomic sequencing. The genes were assigned to the corresponding pathway according to the SCycDB. The total number of sequences was normalized to 42,996 per sample for analysis.

All eight sulfur cycling pathways in the SCycDB (26) were found in all layers of the push core (Fig. 5 and Table S6). There were many genes related to assimilatory sulfate reduction, the link between inorganic and organic sulfur transformation, organic sulfur transformation, and others (transporters for sulfate, sulfite, thiosulfate, and organic sulfur compounds). For the pathway of the link between inorganic and organic sulfur transformation, gene families with high abundance included *cysEKM* and *metABCXYZ*, which link the transformation between organic sulfur compounds and sulfide, as well as *cuyA*, *msmA*, *ssuDE*, *suyAB*, and *xsc*, which link the transformation between organic sulfur compounds and sulfite (27, 28). A high abundance of several gene families involved in the organic sulfur transformation pathway was found in the sediments. The gene family *betABC* is related to the use of sulfate ester choline-O-sulfate (29). The family *dmdABCD*, which codes dimethylsulphoniopropionate (DMSP) degradation, had a high abundance in our sediments. Other gene families with higher abundance include *mdh*, which is related to malate dehydrogenase, as well as *tpa* and *pta*, which are responsible for converting C_2_ sulfonate to sulfoacetaldehyde and the transformation of sulfoacetaldehyde to acetyl-coenzyme A (CoA), respectively (29, 30). In total, gene families in the pathways of dissimilatory sulfur reduction and oxidation, SOX systems, sulfur disproportionation, and sulfur oxidation have lower abundances than other pathways or are even absent from the sediments.

### Methane cycling.

**FIG 6:**
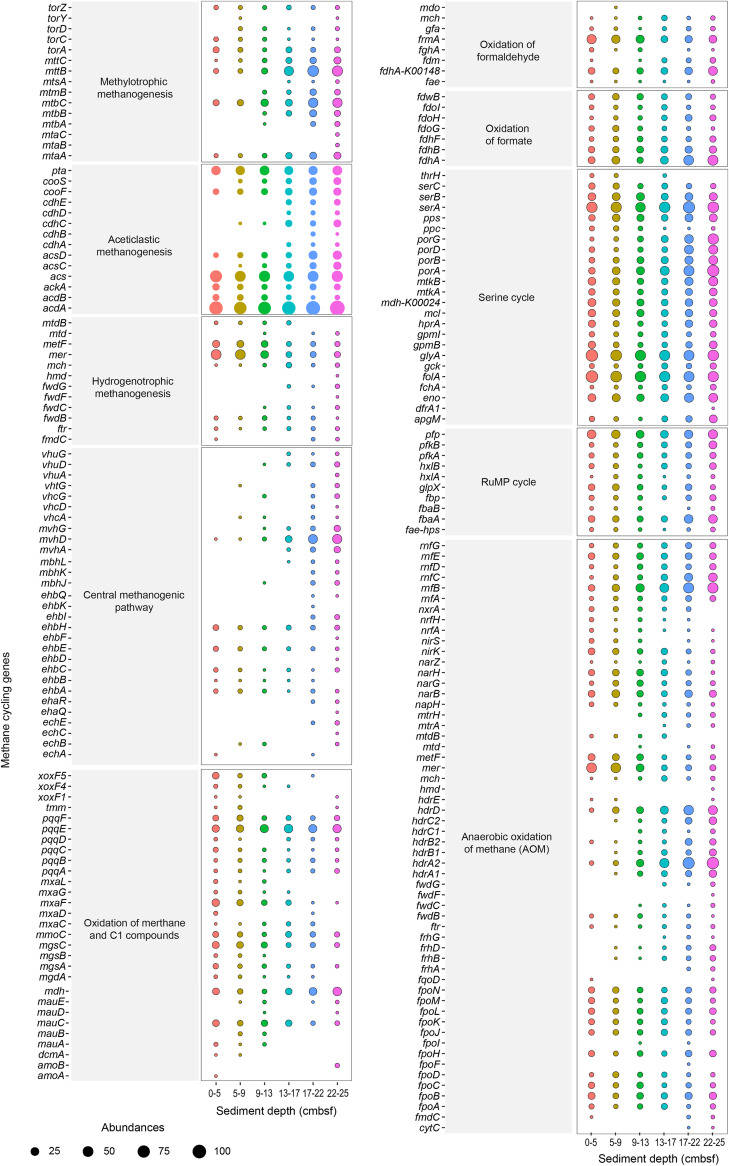
Methane cycling genes in sediment layers of push core F predicted by metagenomic sequencing. The total number of sequences was normalized to 42,996 per sample for analysis.

The gene families covering 10 methane metabolic pathways were predicted to estimate the possibility of methane production and consumption mediated by microorganisms in the sediments (Fig. 6 and Table S7). Methane synthesis can be divided into four major subpathways based on the substrate used (31): hydrogenotrophic methanogenesis, aceticlastic methanogenesis, and methylotrophic methanogenesis, as well as the central methanogenic pathway. Overall, the diversity and abundance of genes associated with methane synthesis were greater at the bottom of the push core than at the top. The number of genes involved in methylotrophic methanogenesis and aceticlastic methanogenesis was greater than that associated with hydrogenotrophic methanogenesis. Gene families (*mttBC* and *mtbABC*) responsible for the oxidation of methyl amines to methane were very abundant, while those for the oxidation of methanol (*mtaABC*) and methyl sulfides (*mtsAB*) were found only in the bottom layer or were even absent from any layer. Aceticlastic methanogenesis activates acetate to acetyl-CoA using either the enzymes acetate kinase/phospho-acetyltransferase (AckA/Pta) or acetyl-CoA synthase (ACS) (32). Then, acetyl-CoA can be used for ATP formation by ADP-forming acetyl-CoA synthase (encoded by *acd*) (33). Gene families (*ackA*, *pta*, *acs*, *acdAB*) that encode these enzymes were abundant along the entire push core, indicating a potentially activated aceticlastic methanogenesis pathway. Almost all gene families involved in hydrogenotrophic methanogenesis were of low abundance, and some key gene families such as *fmdABDFE*, *fwdADHE*, and *mtdAB* were absent from our data set, indicating an incomplete pathway.

Five subpathways involving the aerobic oxidation of methane were observed, and gene families related to these subpathways were abundant in almost every sediment layer. The oxidation of methane and C_1_ compounds constitutes the first two steps of aerobic methane oxidation (34). The gene family *pmoA* (transformation of methane to methanol), which encodes methane monooxygenase and serves as a marker gene for aerobic methane oxidation, was not found in our data set. However, *mmoC*, which also encodes methane monooxygenase, occurred with high abundance in all sediment layers. Several types of methanol dehydrogenase (MDH) were identified, including Xox-MDH and Mxa-MDH for the transformation of methanol to formaldehyde. The *xoxF* gene family mainly exists in the upper layers, while *mxaF* was found in all layers, but its abundance decreased with increasing depth. The second subpathway is involved in the oxidation of formaldehyde. The gene families for the three well-studied pathways, direct oxidation (*fdhA-k00148*), the glutathione (GSH)-linked pathway (*gfa*, *frmB*, *fghA*), and the HMTP/H4F-linked pathway (*fae*, *mtdB*, *mch*), existed in almost all layers of the sediment push core. Oxidation of formate is the third subpathway, and the relevant gene families (*fdhAB*, *fdhF*, *fdoGHI*, *fdwB*) were found in all sediment layers. The fourth subpathway is serine cycling, the key steps of which are catalyzed by serine hydroxymethyltransferase (*glyA*) and glycerate dehydrogenase (*hprA*) (35). Similarly, almost all gene families associated with the final subpathway, the RuMP pathway, were present in each sediment layer.

Anaerobic oxidation of methane (AOM) is always coupled to the reduction of nitrate, sulfate, Fe-Mn (36), and nitrite (37). The gene families related to nitrate-driven (*nrfAH*, *napH*, *narBGHZ*, *nxrA*) and nitrite-driven (*nirKS*) anaerobic methane oxidation were found in almost all sediment layers. The gene families *rnfABCDEG* and *fpo*, encoding ferredoxin:NAD^+^ oxidoreductase (Fno) and F420H2 dehydrogenase were abundant in all sediment layers. The Rnf complex and Fpo are the membrane-bound electron transport systems associated with energy conservation that carry Na^+^ and proton (H^+^), respectively, out of the cell (38). In summary, the sediments contain almost all the gene families required for AOM, except the gene family *mcr*, which encodes methyl-coenzyme M reductase (conversion of methane to CH_3_-CoM).

## DISCUSSION

### The lowest microbial diversity is in the surface layer rather than in deeper layers.

A high diversity of bacteria and archaea has always been observed in the surface sediment, where species capable of thriving in aerobic and anaerobic environments can coexist (39–42). However, our study found that the alpha-diversity index values fluctuated along the sediment depth and the values of the surface layers were significantly lower than those of the deep layers (Fig. 1), which was similar to the hadal samples from the Izu-Ogasawara Trench (43). This trend may be a unique feature of the marine canyon and hadal biosphere, which could be influenced by the varied nutrient supply (i.e., variation in sedimentation rate over a long timescale) (44) or accidental deposition of sediments via landslide. The latest sedimentary geological research in the study area suggests that sediments below 10 cmbsf may have been rapidly deposited by turbidity currents (20), which can supply large amounts of materials and may bring organic matter (45, 46). Shell fragments and bioclastic-rich layers, for instance, were found at the bottom of push cores F and I-2 (another push core next to I1), respectively, indicating that these layers may be rich in organic matter. In contrast, the top sediment layers may have been formed by slow deposition in the absence of recent active turbidite systems (20). Organic matter is consumed by microorganisms in the water during sedimentation, and slow sedimentation gives the microbes more time for metabolism, resulting in fewer nutrients reaching the sediment. Therefore, we proposed that in canyon C4, the nutrient content in the surface sediments should be lower than that in the middle and bottom layers, which is also supported by the recent low deposition rate reflected by the unpublished dating data of the sediments. Diverse and abundant substrates could significantly enhance microbial diversity (47, 48), which may explain why microbial diversity is lowest in surface sediments.

### Greater microbial diversity than in other areas.

The microbial diversity we found is higher than that previously reported in the sediments from other areas of the SCS and the North Chinese Margin Seas (49–51). This indicates a relatively stable microbial system in the study area. The great diversity in our research may be caused by the so-called canyon effect (52). In other words, the biodiversity or biomass in the canyon is always much higher than that of the slope and surrounding areas at a similar depth (53). Moreover, clear color-shifted boundaries were identified along the vertical direction of the push cores (20), indicating different characteristics and sedimentary ages that might support diverse taxa. This result suggests an unexplored microbial resource in the SCS.

### Archaea and bacteria dominated the surface and deep sediment layers, respectively.

Many studies have found that the relative abundance of bacteria decreases with sediment depth, while that of archaea increases with sediment depth (42, 54, 55), and one study showed that archaea make up at least 87% of live cells in sediments buried deeper than 1 m in a wide range of oceanographic settings (56). Unexpectedly, we observed the opposite situation in this study (Fig. S3). We proposed that the opposite distribution trend of bacteria and archaea in terms of relative abundance along depth may be linked to the local environment, such as site-specific geochemical and physical conditions (40), microbial adaptation, bioturbation (57), sediment water content, and grain size (58). Archaea composed 41.04% of the sequences, especially in the surface sediments, which exceeded the proportion of bacteria. A high proportion of archaea was also found in other studies (56, 59, 60). It has been argued that archaea should be better adapted than bacteria to extremely low-energy conditions due to the lower membrane permeability resulting from the lipid structure, which reduces the energy requirement of archaeal cells relative to bacterial cells for maintaining cell integrity under conditions of limited energy availability (61). Surface sediments in this area were most likely formed by slow deposition (20), and sediment dating also supports this view (with a deposition rate of about 0.49 to 1.68 cm/ka, Liao et al. unpublished). Therefore, energy limitation may be an important reason for the high proportion of archaea.

### Community turnover and assembly.

A previous study of mud sediments in the Eastern China Marginal Seas, which cover a large area, found that bacterial communities of the upper layers in each site were separated due to different environmental factors, while deep-layer samples (>20 cmbsf) had a closer clustering relationship regardless of the horizontal sites (42). In our study, even the surface layer samples of different push cores were clustered, which could be due to the shorter geographic distance among the push cores (62). Previous studies in this area have shown that the sediments have similar properties in the horizontal direction, such as the grain size composition (20), suggesting that a similar environment in the horizontal direction leads to the similar community structure. The low proportion of heterogeneous selection between the push cores also supports the hypothesis (Fig. 3d). Other studies have found that differences in microbial community composition among vertical layers of sediment are influenced by the variability of environmental conditions, viral production rates (44), and topography (63). The vertical sediment layers were formed at different ages and events, so the microbial composition in different faults is significantly different. It can be noted that samples from the middle and bottom layers show a mixed pattern on the NMDS plot, indicating similar community composition. The sediments in the middle and bottom layers are considered to have been rapidly deposited by turbidity currents (20), indicating similar sediment properties (as also suggested by the high proportion of homogeneous selection; see Fig. 3c) and age, which provided a homogeneous environment and evolutionary time for microorganisms and then lead to a similar interlayer microbial composition. Conversely, the upper sediments are formed through slow deposition, which may lead to different environmental parameters and evolutionary times. Increased canyon seafloor heterogeneity supports higher species turnover (64), so the difference in microbial community composition between the upper layers is easier to observe (Fig. 3a).

### Elemental cycling.

Our research shows that sedimentary microorganisms in submarine canyons contain many genes related to the cycling of sulfur, carbon, and methane, indicating that submarine canyons have great potential to be hot spots for biogeochemical cycling. Sulfur cycling is closely intertwined with other critical element cycles (carbon, nitrogen, iron, and manganese) (28) and is considered to be the main driving force of seafloor microbial life and the biogeochemical cycle (65–67). The high abundance of assimilatory sulfate reduction gene families in our sample is consistent with the conclusion that sulfate reduction is a major process in marine sediments (67). When oxygen, nitrate/nitrite, iron, and manganese oxides are depleted in the sediment, sulfate respiration becomes the most prominent microbial redox process (28). The high abundance of carbohydrate-active enzymes suggests the potential of the sedimentary microbes to catalyze carbohydrate cycling. Marine sediments represent a large and globally important carbon sink, storing about 2,322 Pg of carbon in the top 1 m (68). When marine sediments and microorganisms are resuspended, the rate of carbon remineralization to carbon dioxide will be accelerated (69). Therefore, the possible environmental and climate risks in marine development should be considered, especially when sediment disturbance is involved. A potentially activated aceticlastic methanogenesis pathway may exist in all sediment layers. The aceticlastic subpathway has been reported to have evolved through the transfer of *ackA* and *pta* genes from cellulolytic clostridia to a methanogenic archaeal family, which is likely to have significantly increased biogenic methane production, with global biogeochemical consequences that may include climate change (32). Although we have no direct evidence that these sedimentary microorganisms actively participate in the biogeochemical cycle, the existence of relevant functional genes indicates that they will play an important role when conditions permit. Notably, viruses are also directly or indirectly involved in elemental cycling in marine sediments (70, 71), which cannot be ignored.

In conclusion, our study has discovered a high diversity of bacteria and archaea in submarine canyon sediments of the northern SCS. Community turnover was more evident in the vertical profile than in the horizontal direction, which appears to be mainly caused by different sedimentation processes. Shotgun-metagenomic sequencing and analysis showed that sedimental bacteria and archaea had a high potential to participate in the cycling of sulfur, carbon, and methane, while nitrogen cycling might be weak. As a case of interdisciplinary biology and geology, this study emphasizes the important role of geological activities and physical processes in shaping the microbial community structure in submarine canyon sediments in addition to conventional physical and chemical factors.

## MATERIALS AND METHODS

### Sampling sites and sampling procedures.

The study area has been described in a previous paper (20). Briefly, the sampling area is in the bending area of a canyon (C4) on the northern continental slope of the SCS. A total of 18 push cores were collected by the manned submersible vehicle *Deep-Sea Warrior* and the supporting vessel *R/V Tansuoyihao* during the TS12-02 expedition on 25 July 2019. This study used seven push cores, and detailed information can be found in Table S1. The push cores H2 and I1 were divided into 5-cm intervals, and F was divided into 4-cm or 5-cm intervals according to different colors along the push core, while the others were divided into 1-cm intervals. As a result, 114 subsamples were obtained, which were numbered according to their depth.

### DNA extraction, PCR amplification, and sequencing.

Extraction and purification of total DNA from the sediment subsamples were performed using the PowerSoil DNA isolation kit (Qiagen) according to the manufacturer’s protocols. DNA extractions were quantified using a Qubit 4 fluorometer (Thermo Fisher Scientific, Waltham, MA, USA). The V6 to V8 hypervariable region of the 16S/18S rRNA gene was amplified by PCR (95°C for 3 min, followed by 25 cycles at 95°C for 30 s, 55°C for 45 s, and 72°C for 90 s, and a final extension at 72°C for 10 min) using the primer set pyroLSSU926F/pyroLSSU1392R (72), which is a slightly modified version of 926wF-1392R that can amplify 84%, 71%, and 91% of rRNA gene sequences from bacteria, archaea, and eukaryotes, respectively (73). The primer pair also produce long amplicons (about 500 bp), which have a high resolution to differentiate taxa. All subsamples were amplified in triplicate, and PCR products were detected by electrophoresis in a 2% (wt/vol) agarose gel. Then, triplicate PCR amplicons were pooled together for library preparation to eliminate the potential for PCR-induced error. Purified amplicons were sequenced on the Illumina Miseq PE300 platform at MAGIGENE company (Guangdong, China). Finally, 60,000 raw reads for each subsample of push core F and 30,000 raw reads for each of the other subsamples were generated. The sequencing data from subsamples D06 and N17 failed to pass the quality inspection and were abandoned. Shotgun-metagenomic sequencing for the six subsamples of push core F was performed using the Illumina NovaSeq 6000 PE150 platform, and 10-Gbp raw reads were generated for each subsample. The amplicon and shotgun-metagenomic sequence data set are available in the Sequence Read Archive (SRA) under the accession numbers PRJNA822765 and SAMN27277534 to SAMN27277645.

### Amplicon sequencing analysis.

Most of the routine analysis of amplicon sequencing was completed with QIIME 2 and its plug-in DADA2 (74, 75), such as sequence quality controlling, feature table and representative sequence generation, taxonomy assignment of representative sequences against the SILVA SSURef NR99 version 138.1 database (21), and phylogenetic tree construction. The taxonomy classifier was trained with the following parameters: –p-f-primer AAACTYAAAKGAATTGRCGG –p-r-primer ACGGGCGGTGWGTRC –p-trunc-len 0 –p-min-length 100 –p-max-length 550. The R package vegan (76) was used for alpha-diversity and beta-diversity analysis. The significant difference in alpha-diversity among push cores or sediment layers was computed using the Tukey HSD test integrated into function alpha_boxplot of the R package amplicon (77). Nonmetric multidimensional scaling (NMDS) was applied based on Bray-Curtis distance by the function metaMDS of the R package vegan to identify the community compositions of all samples. The analysis of similarity (ANOSIM) test was performed to check the significance of NMDS. The heatmap based on Bray-Curtis distance was drawn with the R package pheatmap (78). The community assembly framework based on the null model test was conducted as previously described (48). Scripts used for these analyses can be found at https://github.com/liaochenlanruo/SY179_Canyon/tree/master/Amplicon.

### Shotgun-metagenomic sequencing analysis.

Read quality was assessed and controlled with FastQC (79) and Trimmomatic (80), respectively, and then assembled with metaSPAdes (81). Gene prediction was performed using Prodigal (82) with the parameter -p meta. Carbohydrate-active enzymes and nitrogen cycling genes were predicted by searching the protein sequences against the Carbohydrate-Active enZYmes Database (CAZy) (25) and NCycDB (83), respectively. The sulfur and methane cycling genes were predicted by searching against SCycDB (26) and MCycDB (84), respectively. Bubble plots were generated with the R package ggplot2 (85).
