# Supplementary material for: Microbial Diversity, Community Turnover, and Putative Functions in Submarine Canyon Sediments under the Action of Sedimentary Geology
Source: Microbiol Spectr. 2023 Feb 21;11(2):e04210-22. doi: 10.1128/spectrum.04210-22 (PMC10100816; doi:10.1128/spectrum.04210-22)
Supplement: Supplemental file 1 — Supplemental material. Download spectrum.04210-22-s0001.pdf, PDF file, 1.9 MB [file spectrum.04210-22-s0001.pdf]

Supplementary files for

Microbial diversity, community turnover and putative functions in submarine canyon sediments under the action of sedimentary geology

Hualin Liu [a], Xueyu Cai [a], Kunwen Luo [a], Sihan Chen [a], Ming Su [a, b, c], Jianguo Lu [a, b, c, d#]

[a] School of Marine Sciences, Sun Yat-sen University, Zhuhai 519082, China

[b] Southern Marine Science and Engineering Guangdong Laboratory (Zhuhai), Zhuhai 519080, China

[c] Guangdong Provincial Key Laboratory of Marine Resources and Coastal Engineering, Guangzhou Guangdong, 510275, China

[d] Pearl River Estuary Marine Ecosystem Research Station, Ministry of Education, Zhuhai 519000, China

[#]Corresponding author

Jianguo Lu

Email: lujianguo@mail.sysu.edu.cn

Address: Zhuhai Campus, Sun Yat-Sen University, Tangjiawan, Zhuhai, Guangdong, 519082, P.R.China

Running title: sedimentary geology shapes microbial community

Keywords: South China Sea; Biogeochemical cycle; Ecological processes; Turbidity currents

Supplementary texts

Nitrogen cycling.

Although the number of genes related to nitrogen cycling is much smaller than that of the carbon, sulfur, and methane cycling, diversified pathways were observed (Fig. S6 and Table S8). Organic degradation and synthesis, denitrification, dissimilatory nitrate reduction, and assimilatory nitrate reduction are the most abundant nitrogen cycling pathways, while anammox, Nitrification, and nitrogen fixation have very low abundance. Organic degradation and synthesis mainly consist of genes involved in glutamate biosynthesis （*gs_K00264*, *gs_K00265*, *gs_K00284*, *glsA*, *asnB*） and catabolism (*glnA*, *gdh_K00261, gdh_K00261, gdh_K00262, gdh_K15371*) and nitroalkane catabolism (*nmo*). The key genes involved in denitrification (*nirK*/*nirS*, *norBC*, *nosZ*, and *narGHI*), dissimilatory nitrate reduction (*napAB*, *nirBD,* and *nrfACD*), and assimilatory nitrate reduction (*narB*/*nasAB* and *nirA*) are all observed.

Supplementary tables

Table S1. Detail information on sediment push cores

Push core code	Location	Water depth (m)	Core length (cm)	Interval (cm)
B	111.78972°E, 18.18143°N	1924.1	25	1
D	111.78539°E, 18.18538°N	1917.9	26	1
E	111.78381°E, 18.18788°N	1912.2	25	1
F	111.78204°E, 18.19182°N	1907.4	25	4, 5
H2	111.78353°E, 18.18564°N	1910.5	28	5
I1	111.78415°E, 18.18364°N	1911.4	28	5
N	111.78835°E, 18.17011°N	1895.8	20	1

Table S2 Statistics of the assembled metagenomes.

Metagenomes	contigs	Largest contig (bp)	Total length (bp)	N50 (bp)	GC (%)
F01	18,061	109,452	41,032,984	2,319	58.23
F02	22,827	76,045	49,052,364	2,138	58.27
F03	21,958	86,556	44,832,997	2,024	56.66
F04	40,923	195,123	96,076,190	2,393	54.18
F05	39,042	59,676	86,320,449	2,279	52.37
F06	45,359	69,016	100,681,478	2,263	51.34

Table S3 Number of genes involved in carbohydrate, nitrogen, sulfur and methane cycling. Random subsampling has been performed with the number of sequences 42,996.

Gene	F01	F02	F03	F04	F05	F06	SUM
Sulfur	3,508	3,114	2,667	2,882	3,365	3,624	19,160
CAZy	3,114	2,796	2,576	2,885	3,121	3,290	17,782
Methane	1,536	1,382	1,384	1,595	2,096	2,430	10,423
Nitrogen	501	488	417	440	521	475	2,842
SUM	8,659	7,780	7,044	7,802	9,103	9,819	50,207
Total genes in each assembly	42,996	53,766	49,978	98,679	86,474	99,548	431,441

Table S4 The number of the six CAZy categories in each sample. Random subsampling has been performed with the number of sequences 42,996.

CAZy	F01	F02	F03	F04	F05	F06
AA	144	91	84	61	95	103
CBM	272	237	239	342	283	246
CE	227	240	206	206	250	243
GH	1,132	965	887	1,054	1,111	1,256
GT	1,304	1,237	1,137	1,190	1,352	1,406
PL	35	26	23	32	30	36

Table S5 The number of carbohydrate-active enzymes (CAZymes) in each sample. Random subsampling has been performed with the number of sequences 42,996.

CAZy	F01	F02	F03	F04	F05	F06	Total abundance
AA1_2	3	3	4	1	0	1	12
AA1_3	1	0	0	0	0	0	1
AA10	0	0	0	0	1	1	2
AA12	1	0	0	0	1	0	2
AA15	29	14	11	9	13	18	94
AA17	11	8	8	3	7	8	45
AA2	4	2	0	1	0	1	8
AA3	10	8	7	2	1	0	28
AA3_1	1	0	0	0	1	0	2
AA3_2	39	19	19	15	33	25	150
AA3_3	1	0	0	0	0	0	1
AA4	30	26	24	25	29	38	172
AA5	2	0	4	0	6	8	20
AA5_1	7	6	2	3	0	1	19
AA7	5	4	3	2	3	1	18
AA8	0	1	2	0	0	1	4
CBM0	1	1	1	0	0	0	3
CBM1	1	0	0	0	0	0	1
CBM10	0	0	2	1	2	0	5
CBM11	0	1	1	1	0	0	3
CBM13	48	42	35	44	39	31	239
CBM16	1	1	2	2	1	2	9
CBM18	4	2	1	3	2	3	15
CBM2	24	18	15	17	22	10	106
CBM20	23	19	19	18	15	13	107
CBM22	0	1	0	1	0	0	2
CBM23	0	0	0	0	0	0	0
CBM25	0	0	0	0	1	0	1
CBM27	0	0	0	0	0	0	0
CBM3	3	3	3	3	4	5	21
CBM31	0	0	0	0	0	1	1
CBM32	1	2	5	7	12	8	35
CBM34	0	0	1	3	2	1	7
CBM35	0	0	1	11	8	3	23
CBM38	9	11	15	12	7	9	63
CBM4	1	2	1	3	2	0	9
CBM40	0	0	0	0	0	2	2
CBM41	9	5	8	11	11	6	50
CBM42	2	3	1	1	3	0	10
CBM47	18	17	10	28	18	26	117

CBM48	20	19	24	26	24	26	139
CBM5	0	0	0	1	1	1	3
CBM50	21	14	20	22	21	27	125
CBM51	17	24	22	38	28	23	152
CBM54	1	0	0	0	0	0	1
CBM56	1	1	1	0	0	0	3
CBM57	13	11	10	35	22	16	107
CBM6	32	22	18	23	16	17	128
CBM62	3	0	0	0	0	0	3
CBM64	0	0	0	1	0	0	1
CBM66	9	9	10	19	15	11	73
CBM67	0	1	0	2	1	0	4
CBM70	0	0	0	0	0	0	0
CBM73	5	5	5	5	2	0	22
CBM88	0	0	0	2	1	1	4
CBM9	5	3	8	2	3	4	25
CE0	5	2	3	7	6	3	26
CE1	7	1	2	2	3	1	16
CE11	56	89	81	63	60	63	412
CE12	2	1	0	0	0	1	4
CE15	1	1	0	0	1	0	3
CE16	0	0	0	0	0	0	0
CE3	2	2	1	1	1	2	9
CE4	2	2	3	3	6	8	24
CE5	0	0	0	1	0	0	1
CE6	3	4	2	3	2	6	20
CE7	4	3	2	2	5	6	22
CE8	145	135	112	124	166	153	835
GH0	1	2	2	0	3	2	10
GH1	1	1	1	2	2	10	17
GH10	7	3	11	25	13	9	68
GH100	41	39	24	38	29	41	212
GH102	4	2	3	3	4	6	22
GH103	37	28	27	20	19	26	157
GH104	14	9	8	3	4	9	47
GH105	7	4	6	7	20	38	82
GH106	0	0	0	3	4	3	10
GH108	1	1	6	5	0	0	13
GH109	12	8	14	18	32	38	122
GH11	0	1	0	0	0	2	3
GH110	0	0	0	0	1	1	2
GH113	0	1	0	2	4	1	8
GH114	4	7	9	6	3	1	30
GH115	10	4	1	3	7	2	27

GH116	0	1	0	0	3	3	7
GH117	0	0	0	0	0	0	0
GH120	0	0	0	1	1	1	3
GH123	0	0	0	0	0	1	1
GH125	0	1	0	0	0	0	1
GH126	2	3	0	5	1	4	15
GH128	6	7	3	8	11	14	49
GH129	0	0	0	0	1	0	1
GH13	78	70	51	95	94	85	473
GH13_10	1	0	0	0	0	0	1
GH13_11	54	40	28	31	29	48	230
GH13_13	0	0	1	0	0	0	1
GH13_14	0	0	1	0	1	0	2
GH13_16	1	1	0	1	0	0	3
GH13_17	11	5	6	5	11	6	44
GH13_18	0	1	1	1	1	2	6
GH13_19	0	0	0	0	1	0	1
GH13_2	0	0	0	0	1	0	1
GH13_20	0	1	0	1	0	1	3
GH13_21	0	1	0	0	0	0	1
GH13_23	7	7	11	6	13	11	55
GH13_26	9	11	10	9	18	12	69
GH13_3	1	1	0	1	0	0	3
GH13_30	11	6	15	14	15	17	78
GH13_31	1	0	1	1	0	0	3
GH13_32	1	2	1	0	2	0	6
GH13_33	0	0	0	0	0	1	1
GH13_36	0	0	1	2	1	0	4
GH13_38	0	0	0	0	1	2	3
GH13_39	9	5	7	9	5	9	44
GH13_40	1	0	1	0	1	0	3
GH13_7	0	0	1	2	1	1	5
GH13_8	14	10	6	12	6	8	56
GH13_9	1	5	3	6	9	5	29
GH130	3	7	9	7	13	13	52
GH133	1	1	0	1	1	0	4
GH135	15	8	5	1	7	5	41
GH136	0	1	0	1	1	3	6
GH140	0	2	2	6	6	7	23
GH141	0	0	1	1	0	1	3
GH144	0	0	0	1	1	2	4
GH146	0	0	0	1	1	1	3
GH147	0	0	0	0	0	0	0
GH148	0	0	0	1	0	0	1

GH15	11	14	10	8	11	13	67
GH151	0	0	0	0	0	1	1
GH152	14	19	12	5	4	8	62
GH154	3	0	0	1	0	0	4
GH159	7	5	4	2	2	2	22
GH16	9	8	7	14	17	19	74
GH16_1	1	0	0	0	2	1	4
GH16_14	0	0	0	1	0	0	1
GH16_17	1	1	0	1	1	1	5
GH16_2	0	0	0	1	2	3	6
GH16_20	3	3	3	5	4	5	23
GH16_24	0	0	1	0	1	0	2
GH16_27	7	4	4	5	5	8	33
GH16_3	3	1	2	6	3	5	20
GH16_4	2	1	1	0	0	0	4
GH16_5	0	1	0	0	0	0	1
GH160	0	0	0	0	1	0	1
GH161	0	0	1	0	0	0	1
GH163	0	0	0	0	1	0	1
GH165	0	0	0	1	0	0	1
GH166	0	0	1	0	0	0	1
GH18	2	2	0	4	1	1	10
GH19	15	18	13	15	11	13	85
GH2	12	18	15	23	29	21	118
GH20	29	25	21	34	34	23	166
GH23	145	123	128	131	92	122	741
GH24	1	5	9	3	2	1	21
GH26	1	1	1	1	1	1	6
GH27	8	9	12	13	9	11	62
GH28	0	0	0	1	0	1	2
GH29	2	0	4	3	7	9	25
GH3	6	5	2	2	9	6	30
GH30_1	0	0	1	0	1	2	4
GH30_3	1	1	0	0	0	0	2
GH30_8	0	0	0	1	1	1	3
GH31	0	0	0	0	1	0	1
GH32	5	5	7	11	27	25	80
GH33	38	21	18	28	41	44	190
GH35	20	25	16	15	17	19	112
GH36	23	14	15	16	16	18	102
GH38	28	18	29	32	43	49	199
GH39	14	17	14	10	18	35	108
GH42	0	0	0	1	2	6	9
GH43_1	1	1	0	0	1	1	4

GH43_11	41	26	24	19	27	34	171
GH43_12	4	4	3	5	8	7	31
GH43_13	3	1	1	1	1	0	7
GH43_15	0	0	1	5	1	1	8
GH43_2	0	0	0	0	2	0	2
GH43_21	0	0	0	1	0	0	1
GH43_22	0	0	0	0	1	1	2
GH43_24	2	0	0	2	0	0	4
GH43_26	0	1	1	1	0	0	3
GH43_28	7	8	6	8	9	7	45
GH43_29	2	2	1	3	1	1	10
GH43_3	0	0	0	1	2	2	5
GH43_30	3	1	4	2	2	6	18
GH43_34	0	1	2	2	3	5	13
GH43_35	1	0	1	0	1	0	3
GH43_5	0	0	0	0	1	1	2
GH43_9	0	2	2	1	3	2	10
GH44	0	0	0	1	0	0	1
GH46	1	1	1	1	0	0	4
GH47	0	0	1	0	0	0	1
GH48	0	0	0	0	0	0	0
GH5	9	7	5	7	4	6	38
GH5_1	0	2	0	1	0	0	3
GH5_11	19	18	12	23	15	25	112
GH5_12	0	2	0	0	1	0	3
GH5_13	1	1	0	1	0	3	6
GH5_14	6	6	4	9	9	8	42
GH5_15	3	1	1	1	1	0	7
GH5_18	0	0	0	0	0	0	0
GH5_19	0	3	0	1	0	1	5
GH5_2	0	0	0	1	1	0	2
GH5_22	0	0	0	0	0	0	0
GH5_25	2	1	0	2	0	5	10
GH5_27	25	19	10	10	8	8	80
GH5_29	43	36	16	13	8	5	121
GH5_38	3	0	1	1	1	0	6
GH5_48	0	0	0	0	0	1	1
GH5_7	23	25	25	35	26	27	161
GH5_8	0	1	0	0	0	0	1
GH5_9	8	6	5	4	8	9	40
GH50	0	0	0	0	3	3	6
GH51	0	1	0	3	2	4	10
GH53	0	0	0	1	0	0	1
GH55	24	13	17	24	19	19	116

GH57	11	5	16	10	13	9	64
GH58	0	0	0	0	2	1	3
GH6	15	23	16	9	24	25	112
GH62	0	0	0	1	1	1	3
GH63	5	2	3	4	5	7	26
GH64	0	0	0	0	0	0	0
GH7	0	1	1	0	0	0	2
GH71	2	1	1	0	2	0	6
GH72	11	11	9	7	9	11	58
GH74	7	3	4	3	1	4	22
GH75	0	0	1	0	0	0	1
GH76	23	21	24	22	25	27	142
GH77	0	1	2	1	1	0	5
GH78	29	15	20	19	21	24	128
GH8	2	1	1	0	0	0	4
GH81	0	0	0	0	0	0	0
GH82	0	0	0	0	0	0	0
GH84	1	2	1	0	0	0	4
GH85	0	0	0	2	0	1	3
GH87	0	0	0	0	0	0	0
GH88	1	0	0	1	0	1	3
GH89	0	0	0	0	0	0	0
GH9	4	5	4	5	3	9	30
GH92	8	10	5	10	9	8	50
GH93	2	0	3	1	0	4	10
GH94	0	0	0	0	0	2	2
GH95	0	0	0	0	2	1	3
GH97	0	0	1	3	0	1	5
GH99	3	3	3	7	6	2	24
GT0	0	0	0	1	0	0	1
GT1	3	3	3	2	0	0	11
GT101	0	0	0	0	0	0	0
GT103	0	0	0	0	1	1	2
GT105	8	7	2	10	7	6	40
GT106	38	38	34	37	41	31	219
GT11	0	1	0	0	0	0	1
GT111	14	12	15	8	15	11	75
GT114	0	0	0	0	0	0	0
GT13	4	2	3	2	2	4	17
GT15	1	0	0	0	1	1	3
GT17	0	1	0	1	0	0	2
GT2	89	110	104	117	113	132	665
GT20	29	23	23	13	26	26	140
GT22	52	28	26	22	29	35	192

GT26	0	1	2	3	1	5	12
GT27	0	0	0	1	0	0	1
GT28	83	59	73	66	70	82	433
GT29	15	17	13	12	21	8	86
GT30	29	28	25	22	26	23	153
GT31	19	20	15	18	32	28	132
GT33	13	9	11	10	14	8	65
GT34	2	0	1	0	1	1	5
GT35	63	64	62	68	64	53	374
GT39	0	0	0	0	1	2	3
GT4	602	582	548	588	677	717	3714
GT41	2	3	5	4	0	1	15
GT43	11	10	9	3	5	9	47
GT45	0	0	0	0	1	0	1
GT47	0	0	1	0	1	2	4
GT48	1	0	0	0	0	0	1
GT49	4	6	2	1	3	2	18
GT5	0	6	4	3	5	6	24
GT50	2	0	0	0	1	0	3
GT51	8	9	1	9	5	5	37
GT53	0	0	0	0	0	0	0
GT58	2	1	1	3	2	2	11
GT59	2	0	3	0	0	0	5
GT60	0	1	0	0	0	0	1
GT61	11	10	8	6	3	7	45
GT64	0	0	0	2	0	0	2
GT66	11	7	14	14	11	17	74
GT67	0	0	0	0	0	0	0
GT7	13	16	11	13	20	21	94
GT71	15	11	6	5	3	4	44
GT73	0	0	0	0	2	0	2
GT74	0	0	0	0	0	1	1
GT75	0	0	0	0	0	0	0
GT76	0	0	0	0	0	1	1
GT77	25	26	20	25	24	33	153
GT8	19	16	10	11	22	19	97
GT81	9	10	16	10	17	18	80
GT82	0	1	1	0	0	0	2
GT83	9	10	4	6	7	8	44
GT84	0	0	0	0	1	0	1
GT87	12	6	9	5	6	9	47
GT89	0	0	0	1	1	1	3
GT9	80	80	49	66	69	66	410
GT90	3	3	2	2	1	0	11

GT92	1	0	1	0	0	0	2
PL0	0	0	0	0	0	0	0
PL1	0	0	0	0	1	0	1
PL1_1	11	8	7	7	8	4	45
PL1_2	0	1	0	2	2	2	7
PL1_6	0	0	1	0	0	0	1
PL1_7	1	1	2	1	0	0	5
PL10_1	0	0	0	1	1	3	5
PL11	0	0	0	1	0	2	3
PL11_1	0	0	0	2	1	1	4
PL11_2	0	0	0	0	0	0	0
PL12	2	2	4	4	5	2	19
PL12_3	1	1	0	0	0	1	3
PL14	0	1	0	0	1	1	3
PL14_3	0	0	0	0	1	0	1
PL15	0	0	0	0	0	1	1
PL15_1	0	0	0	0	0	0	0
PL17_1	0	0	0	2	1	5	8
PL2	0	0	0	1	0	2	3
PL2_1	0	0	0	0	0	0	0
PL21_1	0	0	0	0	1	0	1
PL22_1	12	7	2	2	1	0	24
PL26	0	0	0	0	1	0	1
PL3_2	0	0	0	1	0	0	1
PL31	0	0	0	1	1	1	3
PL33_1	0	0	0	0	0	1	1
PL33_2	0	0	0	1	0	0	1
PL35	4	3	1	1	0	1	10
PL38	0	0	1	0	0	0	1
PL39	0	0	0	0	0	1	1
PL4_2	4	2	2	1	3	5	17
PL42	0	0	0	1	0	0	1
PL6	0	0	1	1	1	0	3
PL6_1	0	0	1	1	1	0	3
PL6_2	0	0	0	0	0	0	0
PL6_3	0	0	1	0	0	0	1
PL8	0	0	0	0	0	1	1
PL8_3	0	0	0	0	0	0	0
PL9_1	0	0	0	1	0	1	2
PL9_3	0	0	0	0	0	1	1

Table S6 The number of sulfur cycling genes in each sample. Random subsampling has been performed with the number of sequences 42,996.

Gene	F01	F02	F03	F04	F05	F06	Annotation	Pathways
cysC	172	143	138	122	122	121	Adenylylsulfate kinase	Assimilatory sulfate reduction
cysD	31	28	30	24	24	17	Sulfate adenylyltransferase subunit 2	Assimilatory sulfate reduction
cysH	19	13	10	15	9	11	Phosphoadenosine phosphosulfate reductase	Assimilatory sulfate reduction
cysI	10	10	14	16	30	32	Sulfite reductase (NADPH) hemoprotein beta-component	Assimilatory sulfate reduction
cysJ	75	77	53	72	79	101	Sulfite reductase (NADPH) flavoprotein alpha-component	Assimilatory sulfate reduction
cysN	46	37	29	31	37	47	Sulfate adenylyltransferase subunit 1	Assimilatory sulfate reduction
cysN_cysC	46	39	33	44	60	59	Bifunctional enzyme CysN/CysC	Assimilatory sulfate reduction
cysQ	45	32	22	19	19	25	3'(2'), 5'-bisphosphate nucleotidase	Assimilatory sulfate reduction
nrnA	19	19	14	23	29	38	Bifunctional oligoribonuclease and PAP phosphatase NrnA	Assimilatory sulfate reduction
sir	32	26	24	15	14	14	Sulfite reductase (ferredoxin)	Assimilatory sulfate reduction
aprA	10	16	13	15	16	23	Adenylylsulfate reductase, subunit A	Dissimilatory sulfur reduction and oxidation
aprB	1	1	5	3	3	1	Adenylylsulfate reductase, subunit B	Dissimilatory sulfur reduction and oxidation
dsrA	6	5	7	13	13	18	Dissimilatory sulfite reductase alpha subunit	Dissimilatory sulfur reduction and oxidation
dsrB	3	3	5	5	8	7	Dissimilatory sulfite reductase beta subunit	Dissimilatory sulfur reduction and oxidation
dsrC	0	0	0	0	6	2	Dissimilatory sulfite reductase related protein	Dissimilatory sulfur reduction and oxidation
dsrD	0	0	0	0	1	0	Protein DsrD	Dissimilatory sulfur reduction and oxidation
dsrE	2	2	1	0	0	0	Sulfurtransferase	Dissimilatory sulfur reduction and oxidation
dsrF	2	3	1	0	0	0	Intracellular sulfur oxidation protein	Dissimilatory sulfur reduction and oxidation
dsrH	2	1	1	0	1	0	Intracellular sulfur oxidation protein	Dissimilatory sulfur reduction and oxidation
dsrJ	1	0	2	3	4	0	Protein DsrJ	Dissimilatory sulfur reduction and oxidation
dsrK	0	0	5	7	9	10	Protein DsrK	Dissimilatory sulfur reduction and oxidation
dsrL	0	1	1	3	0	1	Protein DsrL	Dissimilatory sulfur reduction and oxidation

dsrM	2	2	1	3	4	6	Protein DsrM	Dissimilatory sulfur reduction and oxidation
dsrN	3	2	5	1	6	3	Protein DsrN	Dissimilatory sulfur reduction and oxidation
dsrO	0	0	2	0	0	0	Protein DsrO	Dissimilatory sulfur reduction and oxidation
dsrP	0	0	2	2	9	4	Protein DsrP	Dissimilatory sulfur reduction and oxidation
dsrT	0	0	0	1	1	0	Protein DsrT	Dissimilatory sulfur reduction and oxidation
qmoA	2	3	2	6	2	4	Quinone-modifying oxidoreductase, subunit QmoA	Dissimilatory sulfur reduction and oxidation
qmoB	0	1	4	5	0	5	Quinone-modifying oxidoreductase, subunit QmoB	Dissimilatory sulfur reduction and oxidation
qmoC	0	0	1	2	2	4	Quinone-modifying oxidoreductase, subunit QmoC	Dissimilatory sulfur reduction and oxidation
rdsr	0	0	1	0	0	0	Reverse dissimilatory sulfite reductase	Dissimilatory sulfur reduction and oxidation
sat	47	45	35	41	48	58	Sulfate adenylyltransferase	Dissimilatory sulfur reduction and oxidation
cuyA	22	14	13	12	14	16	L-cysteate sulfo-lyase	Link between inorganic and organic sulfur transformation
cysE	36	37	37	50	69	91	Serine O-acetyltransferase	Link between inorganic and organic sulfur transformation
cysK	175	160	126	122	161	155	Cysteine synthase	Link between inorganic and organic sulfur transformation
cysM	59	49	54	55	45	58	Cysteine synthase	Link between inorganic and organic sulfur transformation
hdrA1	1	7	14	20	38	42	heterodisulfide reductase subunit A1	Link between inorganic and organic sulfur transformation
hdrA2	4	4	13	18	50	66	heterodisulfide reductase subunit A2	Link between inorganic and organic sulfur transformation
hdrB1	1	1	3	3	11	16	heterodisulfide reductase subunit B1	Link between inorganic and organic sulfur transformation
hdrB2	2	2	3	11	12	11	heterodisulfide reductase subunit B2	Link between inorganic and organic sulfur transformation
hdrC1	0	0	2	2	2	3	heterodisulfide reductase subunit C1	Link between inorganic and organic sulfur transformation
hdrC2	0	0	1	11	10	21	heterodisulfide reductase subunit C2	Link between inorganic and organic sulfur transformation
hdrD	6	11	13	29	41	42	heterodisulfide reductase subunit D	Link between inorganic and organic sulfur transformation
hdrE	1	1	0	1	0	1	heterodisulfide reductase subunit E	Link between inorganic and organic sulfur transformation

mccB	5	5	7	3	4	0	Cystathionine gamma-lyase / homocysteine desulfhydrase	Link between inorganic and organic sulfur transformation
metA	5	4	2	2	2	3	Homoserine O-succinyltransferase/ O-acetyltransferase	Link between inorganic and organic sulfur transformation
metB	39	40	28	22	48	34	Cystathionine gamma-synthase	Link between inorganic and organic sulfur transformation
metC	66	56	50	60	53	52	Cystathionine beta-lyase	Link between inorganic and organic sulfur transformation
metX	6	16	8	12	15	13	Homoserine O-acetyltransferase/O-succinyltransferase	Link between inorganic and organic sulfur transformation
metY	12	9	5	16	20	17	O-acetyl-L-homoserine sulfhydrylase	Link between inorganic and organic sulfur transformation
metZ	41	48	26	34	37	35	O-succinylhomoserine sulfhydrylase	Link between inorganic and organic sulfur transformation
msmA	13	3	3	1	1	1	Methanesulfonate monooxygenase subunit alpha	Link between inorganic and organic sulfur transformation
mtoX	0	1	1	0	0	0	Methanethiol oxidase	Link between inorganic and organic sulfur transformation
ssuD	73	68	43	32	32	32	Alkanesulfonate monooxygenase	Link between inorganic and organic sulfur transformation
ssuE	13	8	7	6	12	12	FMN reductase	Link between inorganic and organic sulfur transformation
suyA	5	3	5	6	10	8	(2R)-sulfolactate sulfo-lyase subunit alpha	Link between inorganic and organic sulfur transformation
suyB	2	3	0	2	3	5	(2R)-sulfolactate sulfo-lyase subunit beta	Link between inorganic and organic sulfur transformation
tauD	47	27	27	37	21	25	Taurine dioxygenase	Link between inorganic and organic sulfur transformation
tbuB	2	2	0	0	0	0	Toluene-3-monooxygenase	Link between inorganic and organic sulfur transformation
tbuC	8	3	1	0	3	5	Toluene-3-monooxygenase	Link between inorganic and organic sulfur transformation
tmoC	6	5	3	1	1	2	Toluene-4-monooxygenase	Link between inorganic and organic sulfur transformation
touF	1	2	1	1	0	0	Toluene o-xylene monooxygenase	Link between inorganic and organic sulfur transformation
xsc	62	50	31	29	34	33	Sulfoacetaldehyde acetyltransferase	Link between inorganic and organic sulfur transformation
acuI	47	46	38	26	19	17	Acrylyl-CoA reductase AcuI	Organic sulfur transformation
acuK	2	0	0	0	0	0	Enoyl-CoA hydratase/isomerases AcuK	Organic sulfur transformation
acuN	0	3	1	0	0	0	CoA-transferase family III AcuN	Organic sulfur transformation
betA	78	53	49	38	49	71	Oxygen-dependent choline dehydrogenase	Organic sulfur transformation
betB	77	64	56	59	66	76	NAD/NADP-dependent betaine aldehyde dehydrogenase	Organic sulfur transformation

betC	52	27	27	51	52	51	Choline-sulfatase	Organic sulfur transformation
comA	1	1	1	3	5	4	Phosphosulfolactate synthase	Organic sulfur transformation
comB	14	10	10	16	12	11	2-phosphosulfolactate phosphatase	Organic sulfur transformation
comC	9	3	1	5	8	6	L-sulfolactate dehydrogenase	Organic sulfur transformation
comD	6	10	1	4	2	1	Sulfopyruvate decarboxylase subunit alpha	Organic sulfur transformation
comE	12	13	6	13	7	8	Sulfopyruvate decarboxylase subunit beta	Organic sulfur transformation
dddD	3	0	0	0	1	0	CoA-transferase/lyase DddD	Organic sulfur transformation
dddP	39	19	12	19	22	27	Dimethlysulfonioproprionate lyase DddP	Organic sulfur transformation
dddQ	2	1	0	1	0	1	Dimethlysulfonioproprionate lyase DddQ	Organic sulfur transformation
dddT	7	5	2	1	0	0	Betaine/carnitine/choline transporter DddT	Organic sulfur transformation
dddW	1	1	0	1	1	1	Dimethlysulfonioproprionate lyase DddW	Organic sulfur transformation
ddhA	3	4	6	4	7	4	Dimethylsulfide dehydrogenase subunit alpha	Organic sulfur transformation
ddhC	5	3	6	1	1	0	Dimethylsulfide dehydrogenase subunit gamma	Organic sulfur transformation
dmdA	23	20	16	13	4	7	Dimethylsulfoniopropionate demethylase	Organic sulfur transformation
dmdB	53	48	42	39	38	34	3-(methylthio)propionyl---CoA ligase	Organic sulfur transformation
dmdC	83	69	71	79	71	79	3-(methylthio)propanoyl-CoA dehydrogenase	Organic sulfur transformation
dmdD	31	29	28	29	13	16	(methylthio)acryloyl-CoA hydratase	Organic sulfur transformation
dmoA	2	1	1	1	1	0	dimethyl-sulfide monooxygenase	Organic sulfur transformation
dmsA	73	93	78	103	82	85	Anaerobic dimethyl sulfoxide reductase subunit A	Organic sulfur transformation
dmsB	8	12	15	15	18	30	Anaerobic dimethyl sulfoxide reductase subunit B	Organic sulfur transformation
dmsC	17	16	17	12	12	13	Anaerobic dimethyl sulfoxide reductase subunit C	Organic sulfur transformation
dsyB	10	12	7	9	12	4	DsyB	Organic sulfur transformation
gdh	22	24	24	27	30	22	Glutamate dehydrogenase (NADP+)	Organic sulfur transformation
hpsN	6	11	3	8	7	10	Sulfopropanediol 3-dehydrogenase	Organic sulfur transformation
hpsO	10	6	3	3	4	3	R or S-dihydroxypropanesulfonate-2-dehydrogenase	Organic sulfur transformation
hpsP	27	19	18	25	52	62	R or S-dihydroxypropanesulfonate-2-dehydrogenase	Organic sulfur transformation
isfD	47	23	22	20	22	22	Sulfoacetaldehyde reductase	Organic sulfur transformation
mddA	12	5	9	4	2	6	Methanethiol S-methyltransferase	Organic sulfur transformation
mdh	115	130	107	125	155	163	Malate dehydrogenase	Organic sulfur transformation
mtsA	0	1	1	5	8	6	Methylthiol:coenzyme M methyltransferase	Organic sulfur transformation
mtsB	3	10	18	33	50	63	Methylated-thiol--corrinoid protein	Organic sulfur transformation
prpE	40	41	31	24	22	20	Propionate--CoA ligase	Organic sulfur transformation
pta	61	52	52	48	84	90	Phosphate acetyltransferase	Organic sulfur transformation
sfnG	8	6	2	2	0	1	Dimethylsulfone monooxygenase	Organic sulfur transformation
slcC	36	17	26	16	45	48	s-sulfolactate dehydrogenase	Organic sulfur transformation
slcD	6	7	4	3	4	1	Sulfolactate dehydrogenase SlcD	Organic sulfur transformation

sqdB	3	6	13	8	9	9	UDP-sulfoquinovose synthase	Organic sulfur transformation
sqdD	0	0	0	0	0	1	Glycosyltransferase	Organic sulfur transformation
sqdX	36	60	61	54	58	76	Glycogen synthase	Organic sulfur transformation
tauX	0	0	0	0	0	1	Taurine dehydrogenase small subunit	Organic sulfur transformation
tauY	1	0	0	0	0	0	Taurine dehydrogenase large subunit	Organic sulfur transformation
tmm	1	1	0	0	0	0	Trimethylamine monooxygenase	Organic sulfur transformation
toa	5	3	2	2	2	2	Taurine:2-oxoglutarate transaminase	Organic sulfur transformation
tpa	62	54	46	37	46	46	Taurine-pyruvate aminotransferase	Organic sulfur transformation
yihQ	0	0	0	1	2	3	Sulfoquinovosidase	Organic sulfur transformation
cysA	57	39	29	42	36	44	Sulfate/thiosulfate import ATP-binding protein CysA	Others
cysP	29	13	13	15	16	26	Thiosulfate-binding protein	Others
cysU	75	52	58	39	50	60	Sulfate transport system permease protein CysT	Others
cysW	7	7	4	8	4	7	Sulfate transport system permease protein CysW	Others
cysZ	14	10	7	7	3	3	Sulfate transporter CysZ	Others
hpsK	6	3	1	3	7	5	Dihydroxypropanesulfonate transporter	Others
hpsL	5	2	2	2	4	3	Dihydroxypropanesulfonate transporter	Others
hpsM	10	5	6	4	7	6	Dihydroxypropanesulfonate transporter	Others
iseM	33	15	7	17	27	32	Isethionate TRAP transporter	Others
sbp	8	7	10	9	5	8	Sulfate-binding protein	Others
sgpB	7	5	7	3	4	2	Sulfur globule protein CV2	Others
sgpC	10	11	8	6	6	10	Sulfur globule protein CV3	Others
ssuA	80	89	69	95	65	34	Sulfonate transport system substrate-binding protein	Others
ssuB	91	81	65	72	80	72	Sulfonate transport system ATP-binding protein	Others
ssuC	51	24	15	25	27	27	Sulfonate transport system permease protein	Others
sulP	51	71	52	44	62	66	Sulfate permease	Others
tauA	13	18	11	18	11	23	Taurine transport system substrate-binding protein	Others
tauB	22	19	24	15	17	27	Taurine transport system ATP-binding protein	Others
tauC	4	1	0	2	0	3	Taurine transport system permease protein	Others
tauE	0	0	1	0	0	0	Sulfite/organosulfonate exporter	Others
tauZ	1	1	1	3	6	2	Membrane protein TauZ	Others
tusA	26	34	27	36	39	46	Sulfur carrier protein TusA	Others
tusB	10	8	13	10	27	35	tRNA 2-thiouridine synthesizing protein B	Others
tusC	1	0	0	0	0	0	tRNA 2-thiouridine synthesizing protein C	Others
tusD	5	4	7	10	9	13	Sulfurtransferase TusD	Others
tusE	9	8	3	4	4	0	Sulfurtransferase TusE	Others
soxA	3	2	1	2	4	5	L-cysteine S-thiosulfotransferase	SOX systems

soxB	6	6	3	5	7	7	S-sulfosulfanyl-L-cysteine sulfohydrolase	SOX systems
soxC	24	19	12	11	10	8	Sulfane dehydrogenase subunit SoxC	SOX systems
soxD	10	3	2	0	0	1	S-disulfanyl-L-cysteine oxidoreductase SoxD	SOX systems
soxX	33	28	14	11	6	4	L-cysteine S-thiosulfotransferase	SOX systems
soxY	7	2	1	7	7	5	Sulfur-oxidizing protein SoxY	SOX systems
soxZ	6	2	1	1	1	1	Sulfur-oxidizing protein SoxZ	SOX systems
phsA	2	6	4	7	7	1	Thiosulfate reductase	Sulfur disproportionation
phsB	2	3	0	0	0	1	Thiosulfate reductase electron transport protein	Sulfur disproportionation
phsC	0	3	0	0	0	0	Thiosulfate reductase cytochrome b subunit	Sulfur disproportionation
tetH	1	0	0	0	0	0	Tetrathionate hydrolase TetH	Sulfur disproportionation
fccA	3	3	2	0	0	0	Cytochrome subunit of sulfide dehydrogenase	Sulfur oxidation
fccB	2	1	3	3	2	3	Sulfide dehydrogenase [flavocytochrome c] flavoprotein chain	Sulfur oxidation
glpE	126	112	75	88	88	100	Thiosulfate sulfurtransferase	Sulfur oxidation
soeA	5	9	5	7	5	2	Sulfite dehydrogenase (quinone) subunit SoeA	Sulfur oxidation
soeC	2	1	1	0	0	0	Sulfite dehydrogenase (quinone) subunit SoeC	Sulfur oxidation
sorA	4	9	6	1	0	1	Sulfite cytochrome c oxidoreductase-subunitA	Sulfur oxidation
sorB	7	3	1	0	0	0	Sulfite cytochrome c oxidoreductase-subunitB	Sulfur oxidation
sqr	2	3	3	2	2	3	Sulfide:quinone oxidoreductase	Sulfur oxidation
sseA	17	13	11	4	4	5	Thiosulfate sulfurtransferase	Sulfur oxidation
tsdA	19	16	12	6	12	9	Thiosulfate dehydrogenase	Sulfur oxidation
tsdB	18	8	8	1	0	0	Thiosulfate dehydrogenase electron acceptor	Sulfur oxidation
asrA	12	7	7	4	9	18	Anaerobic sulfite reductase subunit A	Sulfur reduction
asrB	3	2	3	9	8	17	Anaerobic sulfite reductase subunit B	Sulfur reduction
asrC	3	5	8	11	17	22	Anaerobic sulfite reductase subunit C	Sulfur reduction
fsr	0	0	0	1	0	1	Sulfite reductase (coenzyme F420)	Sulfur reduction
hydA	0	0	1	1	2	6	Sulfhydrogenase subunit alpha	Sulfur reduction
hydB	0	1	2	0	3	3	Sulfhydrogenase subunit beta (sulfur reductase)	Sulfur reduction
hydD	0	0	1	0	10	15	Sulfhydrogenase subunit delta	Sulfur reduction
otr	3	4	0	0	2	1	Octaheme tetrathionate reductase Otr	Sulfur reduction
psrA	0	0	0	0	2	0	Polysulfide reductase chain A	Sulfur reduction
psrB	0	0	0	0	0	0	Polysulfide reductase chain B	Sulfur reduction
psrC	3	5	0	3	4	4	Polysulfide reductase chain C	Sulfur reduction
shyA	0	1	1	1	4	9	Sulfhydrogenase 2 subunit alpha	Sulfur reduction
shyB	0	0	0	0	0	1	Sulfhydrogenase 2 subunit beta	Sulfur reduction

shyC	6	4	6	5	15	18	Sulfhydrogenase 2 subunit gamma	Sulfur reduction
shyD	0	0	2	1	2	2	Sulfhydrogenase 2 subunit delta	Sulfur reduction
sreA	0	0	0	0	0	0	Sulfur reductase molybdopterin subunit	Sulfur reduction
sudA	54	56	60	71	127	122	Sulfide dehydrogenase subunit alpha	Sulfur reduction
sudB	4	5	2	5	12	13	Sulfide dehydrogenase subunit beta	Sulfur reduction
ttrA	26	26	19	17	30	17	Tetrathionate reductase subunit A	Sulfur reduction
ttrB	20	31	28	31	37	32	Tetrathionate reductase subunit B	Sulfur reduction
ttrC	2	1	1	1	0	2	Tetrathionate reductase subunit C	Sulfur reduction

Table S7 The number of methane cycling genes in each sample. Random subsampling has been performed with the number of sequences 42,996.

Gene	F01	F02	F03	F04	F05	F06	Annotation	Pathway
acdA	97	79	84	97	108	124	Acetate---CoA ligase (ADP-forming)	Aceticlastic methanogenesis
acdB	11	10	9	5	2	4	Acetate---CoA ligase (ADP-forming)	Aceticlastic methanogenesis
ackA	14	9	7	5	11	10	Acetate kinase	Aceticlastic methanogenesis
acs	71	52	56	49	52	57	Acetyl-CoA synthetase	Aceticlastic methanogenesis
acsC	0	1	3	5	13	21	Acetyl-CoA decarbonylase/synthase complex	Aceticlastic methanogenesis
acsD	5	7	9	8	28	30	Acetyl-CoA decarbonylase/synthase complex	Aceticlastic methanogenesis
cdhA	0	0	0	3	2	2	Anaerobic carbon-monoxide dehydrogenase, CODH/ACS complex	Aceticlastic methanogenesis
cdhB	0	0	0	0	3	1	Anaerobic carbon-monoxide dehydrogenase, CODH/ACS complex	Aceticlastic methanogenesis
cdhC	0	1	1	13	7	18	Acetyl-CoA decarbonylase/synthase, CODH/ACS complex	Aceticlastic methanogenesis
cdhD	0	0	0	2	3	3	Acetyl-CoA decarbonylase/synthase, CODH/ACS complex	Aceticlastic methanogenesis
cdhE	0	0	0	7	6	10	Acetyl-CoA decarbonylase/synthase, CODH/ACS complex	Aceticlastic methanogenesis
cooF	9	8	11	16	20	20	Anaerobic carbon-monoxide dehydrogenase	Aceticlastic methanogenesis
cooS	0	2	4	8	16	18	Anaerobic carbon-monoxide dehydrogenase	Aceticlastic methanogenesis
pta	33	32	37	28	30	36	Phosphate acetyltransferase	Aceticlastic methanogenesis
cytC	0	0	0	0	1	1	Multiheme c-type cytochrome	Anaerobic oxidation of methane (AOM)
fmdC	1	0	0	0	2	2	Molybdenum containing formylmethanofuran dehydrogenase	Anaerobic oxidation of methane (AOM)
fpoA	7	4	8	12	3	8	F420H2 dehydrogenase	Anaerobic oxidation of methane (AOM)

fpoB	19	10	16	14	20	21	F420H2 dehydrogenase	Anaerobic oxidation of methane (AOM)
fpoC	14	9	15	6	8	8	F420H2 dehydrogenase	Anaerobic oxidation of methane (AOM)
fpoD	4	12	7	8	3	4	F420H2 dehydrogenase	Anaerobic oxidation of methane (AOM)
fpoF	0	0	0	0	1	0	F420H2 dehydrogenase	Anaerobic oxidation of methane (AOM)
fpoH	12	6	10	8	15	16	F420H2 dehydrogenase	Anaerobic oxidation of methane (AOM)
fpoI	0	0	1	0	1	0	F420H2 dehydrogenase	Anaerobic oxidation of methane (AOM)
fpoJ	9	9	9	14	11	8	F420H2 dehydrogenase	Anaerobic oxidation of methane (AOM)
fpoK	11	9	9	12	6	5	F420H2 dehydrogenase	Anaerobic oxidation of methane (AOM)
fpoL	14	11	11	11	11	16	F420H2 dehydrogenase	Anaerobic oxidation of methane (AOM)
fpoM	13	7	6	14	11	7	F420H2 dehydrogenase	Anaerobic oxidation of methane (AOM)
fpoN	13	14	10	9	11	13	F420H2 dehydrogenase	Anaerobic oxidation of methane (AOM)
fqoD	1	0	0	0	0	1	F420H2: quinone oxidoreductase	Anaerobic oxidation of methane (AOM)
fqoH	0	0	0	0	0	0	F420H2: quinone oxidoreductase	Anaerobic oxidation of methane (AOM)
frhA	0	0	0	0	2	4	Coenzyme F420 hydrogenase	Anaerobic oxidation of methane (AOM)
frhB	0	1	1	4	5	9	Coenzyme F420 hydrogenase	Anaerobic oxidation of methane (AOM)
frhD	0	1	1	2	6	12	Coenzyme F420 hydrogenase	Anaerobic oxidation of methane (AOM)
frhG	0	0	0	1	2	1	Coenzyme F420 hydrogenase	Anaerobic oxidation of methane (AOM)
ftr	2	1	2	3	3	3	Formylmethanofuran--tetrahydromethanopterin, N-formyltransferase	Anaerobic oxidation of methane (AOM)
fwdB	3	2	5	2	3	1	Tungsten containing formylmethanofuran dehydrogenase	Anaerobic oxidation of methane (AOM)
fwdC	0	0	1	2	1	3	Tungsten containing formylmethanofuran dehydrogenase	Anaerobic oxidation of methane (AOM)

fwdF	0	0	0	0	0	1	Tungsten containing formylmethanofuran dehydrogenase	Anaerobic oxidation of methane (AOM)
fwdG	0	0	0	2	1	4	Tungsten containing formylmethanofuran dehydrogenase	Anaerobic oxidation of methane (AOM)
hdrA1	0	1	8	11	13	23	Heterodisulfide reductase	Anaerobic oxidation of methane (AOM)
hdrA2	4	7	22	42	73	77	Heterodisulfide reductase	Anaerobic oxidation of methane (AOM)
hdrB1	0	1	4	10	8	19	Heterodisulfide reductase	Anaerobic oxidation of methane (AOM)
hdrB2	2	1	2	3	18	14	Heterodisulfide reductase	Anaerobic oxidation of methane (AOM)
hdrC1	0	0	2	3	1	5	Heterodisulfide reductase	Anaerobic oxidation of methane (AOM)
hdrC2	0	1	2	6	17	24	Heterodisulfide reductase	Anaerobic oxidation of methane (AOM)
hdrD	4	15	18	28	46	38	Heterodisulfide reductase	Anaerobic oxidation of methane (AOM)
hdrE	1	1	0	0	0	1	Heterodisulfide reductase	Anaerobic oxidation of methane (AOM)
hmd	0	0	0	0	0	1	5,10-methenyltetrahydromethanopterin hydrogenase	Anaerobic oxidation of methane (AOM)
mch	1	1	2	9	2	4	Methenyltetrahydromethanopterin cyclohydrolase	Anaerobic oxidation of methane (AOM)
mer	50	48	25	10	11	12	5,10-methylenetetrahydromethanopterin reductase	Anaerobic oxidation of methane (AOM)
metF	19	18	16	6	6	7	5,10-methylenetetrahydrofolate reductase	Anaerobic oxidation of methane (AOM)
mtd	0	0	1	0	1	2	Methylenetetrahydromethanopterin dehydrogenase	Anaerobic oxidation of methane (AOM)
mtdB	2	2	2	4	0	0	Methylenetetrahydromethanopterin dehydrogenase	Anaerobic oxidation of methane (AOM)
mtrA	0	0	0	1	2	2	Tetrahydromethanopterin S-methyltransferase	Anaerobic oxidation of methane (AOM)
mtrH	0	0	2	6	4	6	Tetrahydromethanopterin S-methyltransferase	Anaerobic oxidation of methane (AOM)
napH	5	3	2	2	2	2	Ferredoxin-type protein NapH	Anaerobic oxidation of methane (AOM)
narB	17	26	15	11	23	19	Nitrate reductase	Anaerobic oxidation of methane (AOM)

narG	4	11	14	6	7	6	Nitrate reductase	Anaerobic oxidation of methane (AOM)
narH	13	16	17	15	9	2	Nitrate reductase	Anaerobic oxidation of methane (AOM)
narZ	1	1	1	4	1	2	Nitrate reductase	Anaerobic oxidation of methane (AOM)
nirK	17	13	9	15	6	4	Nitrite reductase (NO-forming)	Anaerobic oxidation of methane (AOM)
nirS	6	3	2	0	1	1	Nitrite reductase (NO-forming)	Anaerobic oxidation of methane (AOM)
nod	0	0	0	0	0	0	NO dismutase	Anaerobic oxidation of methane (AOM)
nrfA	3	2	2	1	1	1	Nitrite reductase (cytochrome c?552)	Anaerobic oxidation of methane (AOM)
nrfH	4	1	3	1	1	0	Cytochrome c-type protein NrfH	Anaerobic oxidation of methane (AOM)
nxrA	6	7	4	3	5	0	Nitrite oxidoreductase	Anaerobic oxidation of methane (AOM)
rnfA	3	5	6	8	11	11	Na+-translocating ferredoxin:NAD+oxidoreductase	Anaerobic oxidation of methane (AOM)
rnfB	15	22	31	39	53	56	Na+-translocating ferredoxin:NAD+oxidoreductase	Anaerobic oxidation of methane (AOM)
rnfC	2	7	7	15	29	35	Na+-translocating ferredoxin:NAD+oxidoreductase	Anaerobic oxidation of methane (AOM)
rnfD	4	3	9	10	9	14	Na+-translocating ferredoxin:NAD+oxidoreductase	Anaerobic oxidation of methane (AOM)
rnfE	16	13	17	13	16	16	Na+-translocating ferredoxin:NAD+oxidoreductase	Anaerobic oxidation of methane (AOM)
rnfG	4	7	7	10	12	12	Na+-translocating ferredoxin:NAD+oxidoreductase	Anaerobic oxidation of methane (AOM)
echA	1	0	0	0	1	0	Ech hydrogenase	Central methanogenic pathway
echB	0	1	2	0	0	2	Ech hydrogenase	Central methanogenic pathway
echC	0	0	0	0	0	1	Ech hydrogenase	Central methanogenic pathway
echE	0	0	0	0	2	4	Ech hydrogenase	Central methanogenic pathway
ehaQ	0	0	0	0	0	1	Energy-converting hydrogenase A	Central methanogenic pathway
ehaR	0	0	0	0	2	2	Energy-converting hydrogenase A	Central methanogenic pathway
ehbA	4	4	3	1	3	2	Energy-converting hydrogenase B	Central methanogenic pathway

ehbB	1	1	1	1	1	0	Energy-converting hydrogenase B	Central methanogenic pathway
ehbC	4	2	1	2	1	4	Energy-converting hydrogenase B	Central methanogenic pathway
ehbD	0	0	0	0	0	1	Energy-converting hydrogenase B	Central methanogenic pathway
ehbE	7	4	3	3	3	2	Energy-converting hydrogenase B	Central methanogenic pathway
ehbF	0	0	0	0	0	1	Energy-converting hydrogenase B	Central methanogenic pathway
ehbH	10	4	3	6	5	6	Energy-converting hydrogenase B	Central methanogenic pathway
ehbI	0	0	0	0	3	6	Energy-converting hydrogenase B	Central methanogenic pathway
ehbK	0	0	0	0	1	0	Energy-converting hydrogenase B	Central methanogenic pathway
ehbQ	0	0	0	0	1	1	Energy-converting hydrogenase B	Central methanogenic pathway
fpoA	7	4	8	12	3	8	F420H2 dehydrogenase	Central methanogenic pathway
fpoB	19	10	16	14	20	21	F420H2 dehydrogenase	Central methanogenic pathway
fpoC	14	9	15	6	8	8	F420H2 dehydrogenase	Central methanogenic pathway
fpoD	4	12	7	8	3	4	F420H2 dehydrogenase	Central methanogenic pathway
fpoF	0	0	0	0	1	0	F420H2 dehydrogenase	Central methanogenic pathway
fpoH	12	6	10	8	15	16	F420H2 dehydrogenase	Central methanogenic pathway
fpoI	0	0	1	0	1	0	F420H2 dehydrogenase	Central methanogenic pathway
fpoJ	9	9	9	14	11	8	F420H2 dehydrogenase	Central methanogenic pathway
fpoK	11	9	9	12	6	5	F420H2 dehydrogenase	Central methanogenic pathway
fpoL	14	11	11	11	11	16	F420H2 dehydrogenase	Central methanogenic pathway
fpoM	13	7	6	14	11	7	F420H2 dehydrogenase	Central methanogenic pathway
fpoN	13	14	10	9	11	13	F420H2 dehydrogenase	Central methanogenic pathway
fqoD	1	0	0	0	0	1	F420H2: quinone oxidoreductase	Central methanogenic pathway

fqoH	0	0	0	0	0	0	F420H2: quinone oxidoreductase	Central methanogenic pathway
frhA	0	0	0	0	2	4	Coenzyme F420 hydrogenase	Central methanogenic pathway
frhB	0	1	1	4	5	9	Coenzyme F420 hydrogenase	Central methanogenic pathway
frhD	0	1	1	2	6	12	Coenzyme F420 hydrogenase	Central methanogenic pathway
frhG	0	0	0	1	2	1	Coenzyme F420 hydrogenase	Central methanogenic pathway
hdrA1	0	1	8	11	13	23	Heterodisulfide reductase	Central methanogenic pathway
hdrA2	4	7	22	42	73	77	Heterodisulfide reductase	Central methanogenic pathway
hdrB1	0	1	4	10	8	19	Heterodisulfide reductase	Central methanogenic pathway
hdrB2	2	1	2	3	18	14	Heterodisulfide reductase	Central methanogenic pathway
hdrC1	0	0	2	3	1	5	Heterodisulfide reductase	Central methanogenic pathway
hdrC2	0	1	2	6	17	24	Heterodisulfide reductase	Central methanogenic pathway
hdrD	4	15	18	28	46	38	Heterodisulfide reductase	Central methanogenic pathway
hdrE	1	1	0	0	0	1	Heterodisulfide reductase	Central methanogenic pathway
mbhJ	0	0	1	0	4	5	Membrane-bound hydrogenase	Central methanogenic pathway
mbhK	0	0	0	0	2	2	Membrane-bound hydrogenase	Central methanogenic pathway
mbhL	0	0	0	1	2	2	Membrane-bound hydrogenase	Central methanogenic pathway
mtrA	0	0	0	1	2	2	Tetrahydromethanopterin S-methyltransferase	Central methanogenic pathway
mtrH	0	0	2	6	4	6	Tetrahydromethanopterin S-methyltransferase	Central methanogenic pathway
mvhA	0	0	0	2	7	14	F420-nonreducing hydrogenase	Central methanogenic pathway
mvhD	1	1	3	17	39	42	F420-nonreducing hydrogenase	Central methanogenic pathway
mvhG	0	0	1	2	5	14	F420-nonreducing hydrogenase	Central methanogenic pathway
rnfA	3	5	6	8	11	11	Na+-translocating ferredoxin:NAD+oxidoreductase	Central methanogenic pathway

rnfB	15	22	31	39	53	56	Na+-translocating ferredoxin:NAD+oxidoreductase	Central methanogenic pathway
rnfC	2	7	7	15	29	35	Na+-translocating ferredoxin:NAD+oxidoreductase	Central methanogenic pathway
rnfD	4	3	9	10	9	14	Na+-translocating ferredoxin:NAD+oxidoreductase	Central methanogenic pathway
rnfE	16	13	17	13	16	16	Na+-translocating ferredoxin:NAD+oxidoreductase	Central methanogenic pathway
rnfG	4	7	7	10	12	12	Na+-translocating ferredoxin:NAD+oxidoreductase	Central methanogenic pathway
vhcA	0	1	1	0	2	1	F420-nonreducing hydrogenase	Central methanogenic pathway
vhcD	0	0	0	0	2	1	F420-nonreducing hydrogenase	Central methanogenic pathway
vhcG	0	0	2	0	2	5	F420-nonreducing hydrogenase	Central methanogenic pathway
vhtG	0	1	0	0	3	4	Methanophenazine hydrogenase	Central methanogenic pathway
vhuA	0	0	0	0	0	2	F420-nonreducing hydrogenase	Central methanogenic pathway
vhuD	0	0	1	2	5	7	F420-nonreducing hydrogenase	Central methanogenic pathway
vhuG	0	0	0	2	1	2	F420-nonreducing hydrogenase	Central methanogenic pathway
fmdC	1	0	0	0	2	2	Molybdenum containing formylmethanofuran dehydrogenase	Hydrogenotrophic methanogenesis
ftr	2	1	2	3	3	3	Formylmethanofuran--tetrahydromethanopterin, N-formyltransferase	Hydrogenotrophic methanogenesis
fwdB	3	2	5	2	3	1	Tungsten containing formylmethanofuran dehydrogenase	Hydrogenotrophic methanogenesis
fwdC	0	0	1	2	1	3	Tungsten containing formylmethanofuran dehydrogenase	Hydrogenotrophic methanogenesis
fwdF	0	0	0	0	0	1	Tungsten containing formylmethanofuran dehydrogenase	Hydrogenotrophic methanogenesis
fwdG	0	0	0	2	1	4	Tungsten containing formylmethanofuran dehydrogenase	Hydrogenotrophic methanogenesis

hmd	0	0	0	0	0	1	5,10-methenyltetrahydromethanopterin hydrogenase	Hydrogenotrophic methanogenesis
mch	1	1	2	9	2	4	Methenyltetrahydromethanopterin cyclohydrolase	Hydrogenotrophic methanogenesis
mer	50	48	25	10	11	12	5,10-methylenetetrahydromethanopterin reductase	Hydrogenotrophic methanogenesis
metF	19	18	16	6	6	7	5,10-methylenetetrahydrofolate reductase	Hydrogenotrophic methanogenesis
mtd	0	0	1	0	1	2	Methylenetetrahydromethanopterin dehydrogenase	Hydrogenotrophic methanogenesis
mtdB	2	2	2	4	0	0	Methylenetetrahydromethanopterin dehydrogenase	Hydrogenotrophic methanogenesis
mtaA	3	3	5	14	18	19	Coenzyme M methyltransferase	Methylotrophic methanogenesis
mtaB	0	0	0	0	0	2	Coenzyme M methyltransferase	Methylotrophic methanogenesis
mtaC	0	0	0	0	0	2	Coenzyme M methyltransferase	Methylotrophic methanogenesis
mtbA	0	0	1	0	6	7	Dimethylamine methyltransferase	Methylotrophic methanogenesis
mtbB	0	0	2	9	19	12	Dimethylamine methyltransferase	Methylotrophic methanogenesis
mtbC	12	13	22	18	44	44	Dimethylamine methyltransferase	Methylotrophic methanogenesis
mtmB	0	0	3	4	14	10	Monomethylamine methyltransferase	Methylotrophic methanogenesis
mtsA	0	0	0	1	1	2	Methylated-thiol--coenzyme m methyltransferase	Methylotrophic methanogenesis
mttB	6	5	12	41	63	56	Trimethylamine methyltransferase	Methylotrophic methanogenesis
mttC	1	2	5	12	14	19	Trimethylamine methyltransferase	Methylotrophic methanogenesis
torA	12	6	3	11	6	11	Trimethylamine-N-oxide reductase	Methylotrophic methanogenesis
torC	5	3	3	1	1	1	Trimethylamine-N-oxide reductase	Methylotrophic methanogenesis
torD	0	3	3	1	2	3	Trimethylamine-N-oxide reductase	Methylotrophic methanogenesis
torY	0	1	0	0	0	1	Trimethylamine-N-oxide reductase	Methylotrophic methanogenesis
torZ	4	3	3	5	3	3	Trimethylamine-N-oxide reductase	Methylotrophic methanogenesis

fae	1	1	1	1	1	2	5,6,7,8-tetrahydromethanopterin hydro-lyase	Oxidation of formaldehyde
fdhA-K00148	16	11	14	17	23	38	Glutathione-independent formaldehyde dehydrogenase	Oxidation of formaldehyde
fdm	1	0	1	7	6	10	Formaldehyde dismutase	Oxidation of formaldehyde
fghA	4	1	3	0	1	1	S-formylglutathione hydrolase	Oxidation of formaldehyde
frmA	41	31	29	17	21	25	S-(hydroxymethyl)glutathione dehydrogenase/alcohol dehydrogenase	Oxidation of formaldehyde
gfa	2	2	1	3	5	5	S-(hydroxymethyl)glutathione synthase	Oxidation of formaldehyde
mch	1	1	2	9	2	4	Methenyltetrahydromethanopterin cyclohydrolase	Oxidation of formaldehyde
mdo	0	1	0	0	0	0	Formaldehyde dismutase / methanol dehydrogenase	Oxidation of formaldehyde
fdhA	18	26	13	26	49	53	Formate dehydrogenase (coenzyme F420/NADP+)	Oxidation of formate
fdhB	14	14	17	19	20	21	Formate dehydrogenase (coenzyme F420/NADP+)	Oxidation of formate
fdhF	5	9	10	10	8	13	Formate dehydrogenase H	Oxidation of formate
fdoG	8	13	6	5	3	2	Formate dehydrogenase	Oxidation of formate
fdoH	2	4	4	9	16	12	Formate dehydrogenase	Oxidation of formate
fdoI	5	4	4	9	4	5	Formate dehydrogenase	Oxidation of formate
fdwB	11	15	14	11	11	7	Tungsten-containing formate dehydrogenase	Oxidation of formate
amoA	1	0	0	0	0	0	Ammonia monooxygenase	Oxidation of merthane and C1 compounds
amoB	0	0	0	0	0	4	Ammonia monooxygenase	Oxidation of merthane and C1 compounds
dcmA	1	1	0	0	0	0	Transcriptional repressor of dcmA and dcmR	Oxidation of merthane and C1 compounds
dcmR	0	0	0	0	0	0	Dichloromethane dehalogenase	Oxidation of merthane and C1 compounds
mauA	3	1	2	0	0	0	Methylamine dehydrogenase	Oxidation of merthane and C1 compounds
mauB	0	3	2	0	0	0	Methylamine dehydrogenase	Oxidation of merthane and C1 compounds
mauC	16	9	14	14	4	5	Methylamine dehydrogenase	Oxidation of merthane and C1 compounds
mauD	0	0	1	0	0	1	Methylamine dehydrogenase	Oxidation of merthane and C1 compounds

mauE	0	1	2	0	1	2	Methylamine dehydrogenase	Oxidation of merthane and C1 compounds
mdh	17	8	5	15	25	35	NAD-dependent methanol dehydrogenase	Oxidation of merthane and C1 compounds
mgdA	2	2	1	1	1	0	Methylglutamate dehydrogenase	Oxidation of merthane and C1 compounds
mgsA	8	4	4	3	2	2	Methylamine---glutamate N-methyltransferase	Oxidation of merthane and C1 compounds
mgsB	2	2	1	0	0	0	Methylamine---glutamate N-methyltransferase	Oxidation of merthane and C1 compounds
mgsC	18	16	12	6	7	6	Methylamine---glutamate N-methyltransferase	Oxidation of merthane and C1 compounds
mmoC	11	8	4	11	8	7	Methane monooxygenase	Oxidation of merthane and C1 compounds
mxaC	1	1	2	4	3	0	Methanol dehydrogenase	Oxidation of merthane and C1 compounds
mxaD	3	0	0	0	1	0	Methanol dehydrogenase	Oxidation of merthane and C1 compounds
mxaF	23	13	9	8	1	1	Methanol dehydrogenase	Oxidation of merthane and C1 compounds
mxaG	2	2	1	1	0	0	Methanol dehydrogenase	Oxidation of merthane and C1 compounds
mxaL	3	1	2	0	0	0	Methanol dehydrogenase	Oxidation of merthane and C1 compounds
pqqA	2	2	1	2	3	5	Coenzyme PQQ synthesis	Oxidation of merthane and C1 compounds
pqqB	2	3	3	2	4	2	Coenzyme PQQ synthesis	Oxidation of merthane and C1 compounds
pqqC	6	2	2	1	1	2	Coenzyme PQQ synthesis	Oxidation of merthane and C1 compounds
pqqD	2	1	0	2	2	2	Coenzyme PQQ synthesis	Oxidation of merthane and C1 compounds
pqqE	21	21	29	33	29	31	Coenzyme PQQ synthesis	Oxidation of merthane and C1 compounds
pqqF	10	12	5	6	5	8	Coenzyme PQQ synthesis	Oxidation of merthane and C1 compounds
tmm	1	3	0	0	0	1	Trimethylamine monooxygenase	Oxidation of merthane and C1 compounds
xoxF1	1	1	0	0	0	1	Methanol dehydrogenase	Oxidation of merthane and C1 compounds
xoxF4	3	2	1	1	0	0	Methanol dehydrogenase	Oxidation of merthane and C1 compounds
xoxF5	16	4	5	0	1	0	Methanol dehydrogenase	Oxidation of merthane and C1 compounds

fae-hps	3	3	2	1	2	5	Bifunctional enzyme Fae/Hps	RuMP cycle
fbaA	16	14	12	13	34	35	Fructose-bisphosphate aldolase	RuMP cycle
fbaB	2	2	2	0	1	3	Fructose-bisphosphate aldolase	RuMP cycle
fbp	7	5	6	3	9	13	Fructose?1,6-bisphosphatase I	RuMP cycle
glpX	15	16	10	5	15	7	Fructose?1,6-bisphosphatase II	RuMP cycle
hxlA	1	4	4	1	2	3	3-hexulose?6-phosphate synthase	RuMP cycle
hxlB	7	7	3	10	8	16	6-phospho?3-hexuloisomerase	RuMP cycle
pfkA	7	7	10	7	10	13	6-phosphofructokinase	RuMP cycle
pfkB	5	5	8	11	12	27	6-phosphofructokinase	RuMP cycle
pfp	35	32	26	25	31	41	Pyrophosphate--fructose 6-phosphate 1-phosphotransferase	RuMP cycle
apgM	8	5	2	11	14	27	2,3-bisphosphoglycerate-independent phosphoglycerate mutase	Serine cycle
dfrA1	0	0	0	0	0	1	Dihydrofolate reductase	Serine cycle
eno	26	27	22	27	40	38	Enolase	Serine cycle
fchA	2	3	3	8	5	11	Methenyltetrahydrofolate cyclohydrolase	Serine cycle
folA	80	64	60	58	54	57	Dihydrofolate reductase	Serine cycle
gck	6	4	6	12	8	13	Glycerate 2-kinase	Serine cycle
glyA	79	63	57	56	53	61	Glycine hydroxymethyltransferase	Serine cycle
gpmB	21	10	7	9	14	25	Phosphoglycerate mutase	Serine cycle
gpmI	8	6	6	12	6	9	Phosphoglycerate mutase	Serine cycle
hprA	20	12	8	9	22	23	Glycerate dehydrogenase	Serine cycle
mcl	23	19	21	18	22	20	Malyl-CoA/(S)-citramalyl-CoA lyase	Serine cycle
mdh-K00024	27	21	19	16	24	26	Malate dehydrogenase	Serine cycle
mtkA	15	12	10	12	18	25	Malate-CoA ligase	Serine cycle
mtkB	25	20	14	15	30	41	Malate-CoA ligase	Serine cycle
porA	15	25	28	39	54	74	Pyruvate ferredoxin oxidoreductase	Serine cycle
porB	10	12	18	20	30	38	Pyruvate ferredoxin oxidoreductase	Serine cycle
porD	9	4	8	10	37	40	Pyruvate ferredoxin oxidoreductase	Serine cycle
porG	3	5	11	18	35	58	Pyruvate ferredoxin oxidoreductase	Serine cycle
ppc	6	8	5	1	1	2	Phosphoenolpyruvate carboxylase	Serine cycle
pps	14	23	20	16	27	27	Pyruvate, water dikinase	Serine cycle
serA	59	56	46	59	74	62	D?3-phosphoglycerate dehydrogenase / 2-oxoglutarate reductase	Serine cycle
serB	25	26	28	18	26	28	Phosphoserine phosphatase	Serine cycle

serC	14	13	8	11	8	8	Phosphoserine aminotransferase	Serine cycle
thrH	3	3	0	2	0	0	Phosphoserine / homoserine phosphotransferase	Serine cycle

Table S8 The number of nitrogen cycling genes in each sample. Random subsampling has been performed with the number of sequences 42,996.

Gene	F01	F02	F03	F04	F05	F06	Annotation	Pathways
hdh	0	2	0	2	1	0	Hydrazine dehydrogenase	Anammox
hzo	0	2	5	0	1	0	Hydrazine oxidoreductase	Anammox
hzsA	0	2	1	5	2	1	Hydrazine synthase subunit A	Anammox
hzsB	0	2	0	2	1	0	Hydrazine synthase subunit B	Anammox
hzsC	0	1	2	1	1	0	Hydrazine synthase subunit C	Anammox
amoA_B	1	0	0	0	0	0	Ammonia monooxygenase subunit A (bacteria)	Nitrification
amoB_A	1	0	0	0	0	0	Ammonia monooxygenase subunit B (archaea)	Nitrification
amoC_A	0	0	0	0	0	0	Ammonia monooxygenase subunit C (archaea)	Nitrification
hao	2	6	2	7	9	9	Hydroxylamine dehydrogenase	Nitrification
nxrA	0	2	1	0	0	0	Nitrite oxidoreductase, alpha subunit	Nitrification
nxrB	2	4	5	2	1	0	Nitrite oxidoreductase, beta subunit	Nitrification
narG	8	15	12	5	9	1	Nitrate reductase	Denitrification
narH	11	16	14	12	6	2	Nitrate reductase	Denitrification
narI	1	4	1	4	2	4	Nitrate reductase gamma subunit	Denitrification
narJ	8	5	6	4	3	1	Nitrate reductase molybdenum cofactor assembly chaperone	Denitrification
narZ	0	1	1	2	1	2	Nitrate reductase 2, alpha subunit	Denitrification
nirK	15	9	8	8	2	3	Nitrite reductase (NO-forming)	Denitrification
nirS	9	9	11	15	7	10	Nitrite reductase (NO-forming)	Denitrification
norB	0	4	5	2	3	1	Nitric oxide reductase subunit B	Denitrification
norC	3	5	8	5	0	0	Nitric oxide reductase subunit C	Denitrification
nosZ	22	23	19	20	22	9	Nitrous-oxide reductase	Denitrification
narB	11	15	9	10	6	8	Assimilatory nitrate reductase	Assimilatory nitrate reduction
nasA	24	22	16	13	24	13	Assimilatory nitrate reductase catalytic subunit	Assimilatory nitrate reduction
nasB	2	3	1	1	1	2	Assimilatory nitrate reductase electron transfer subunit	Assimilatory nitrate reduction
narC	1	0	0	5	2	1	Cytochrome b-561	Assimilatory nitrate reduction
nirA	9	5	5	4	9	6	Ferredoxin-nitrite reductase	Assimilatory nitrate reduction
NR	12	12	11	7	12	9	Nitrate reductase (NAD(P)H)	Assimilatory nitrate reduction
nrfA	3	2	2	2	3	3	Nitrite reductase (cytochrome c-552)	Dissimilatory nitrate reduction

nirB	7	14	7	14	13	12	Nitrite reductase (NADH) large subunit	Dissimilatory nitrate reduction
nirD	12	7	4	7	4	1	Nitrite reductase (NADH) small subunit	Dissimilatory nitrate reduction
napA	5	10	10	3	15	13	Periplasmic nitrate reductase NapA	Dissimilatory nitrate reduction
napB	1	1	1	1	2	1	Periplasmic nitrate reductase NapB	Dissimilatory nitrate reduction
napC	6	5	3	0	0	0	Periplasmic nitrate reductase NapC	Dissimilatory nitrate reduction
nrfC	11	15	13	25	34	35	Protein NrfC	Dissimilatory nitrate reduction
nrfD	3	5	5	7	2	1	Protein NrfD	Dissimilatory nitrate reduction
nifH	0	0	1	2	9	8	Nitrogenase iron protein NifH	Nitrogen fixation
nifD	0	0	0	0	1	0	Nitrogenase molybdenum-iron protein alpha chain	Nitrogen fixation
nifK	0	1	0	5	5	4	Nitrogenase molybdenum-iron protein beta chain	Nitrogen fixation
asnB	19	21	24	16	22	12	Asparagine synthase (glutamine-hydrolysing)	Organic degradation and synthesis
gdh_K00260	1	1	1	2	0	1	Glutamate dehydrogenase	Organic degradation and synthesis
gdh_K15371	21	17	9	7	9	11	Glutamate dehydrogenase	Organic degradation and synthesis
gdh_K00261	13	16	15	11	20	21	Glutamate dehydrogenase (NAD(P)+)	Organic degradation and synthesis
gdh_K00262	10	6	11	7	9	13	Glutamate dehydrogenase (NADP+)	Organic degradation and synthesis
gs_K00284	6	5	4	1	2	0	Glutamate synthase (ferredoxin)	Organic degradation and synthesis
gs_K00264	0	2	1	1	0	0	Glutamate synthase (NADPH/NADH)	Organic degradation and synthesis
gs_K00265	7	6	7	5	4	1	Glutamate synthase (NADPH/NADH) large chain	Organic degradation and synthesis
gs_K00266	48	51	50	75	127	126	Glutamate synthase (NADPH/NADH) small chain	Organic degradation and synthesis
ansB	4	2	2	2	3	3	Glutamin-(asparagin-)ase	Organic degradation and synthesis
glsA	21	20	11	16	16	15	Glutaminase	Organic degradation and

								synthesis
glnA	102	61	46	37	35	44	Glutamine synthetase	Organic degradation and synthesis
nmo	49	43	37	41	49	50	Nitronate monooxygenase	Organic degradation and synthesis
ureC	9	6	8	8	4	10	Urease subunit alpha	Organic degradation and synthesis
ureB	0	0	0	0	0	1	Urease subunit beta	Organic degradation and synthesis
ureA	1	1	0	1	1	0	Urease subunit gamma	Organic degradation and synthesis
hcp	0	0	2	7	6	7	Hydroxylamine reductase	Others
pmoB	0	1	0	1	1	0	Particulate methane monooxygenase subunit B	Others

Supplementary figures

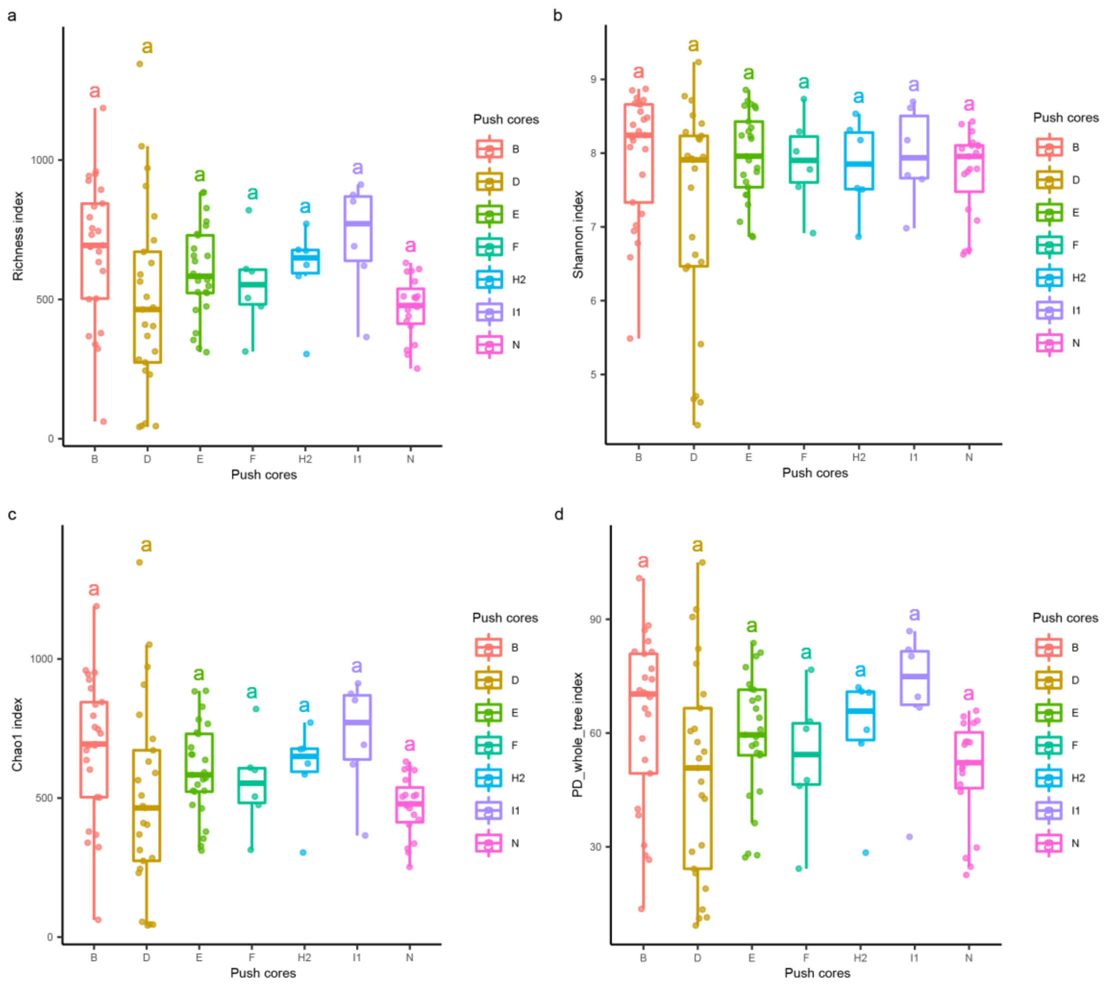

Fig. S1 Comparison of diversity between different sediment cores. (a) richness, (b) Shannon index, (c) Chao 1 index, and (d) phylogenetic diversity. Significant differences between cores are indicated by lowercase letters (adjusted $p < 0.05$, ANOVA, Tukey HSD test).

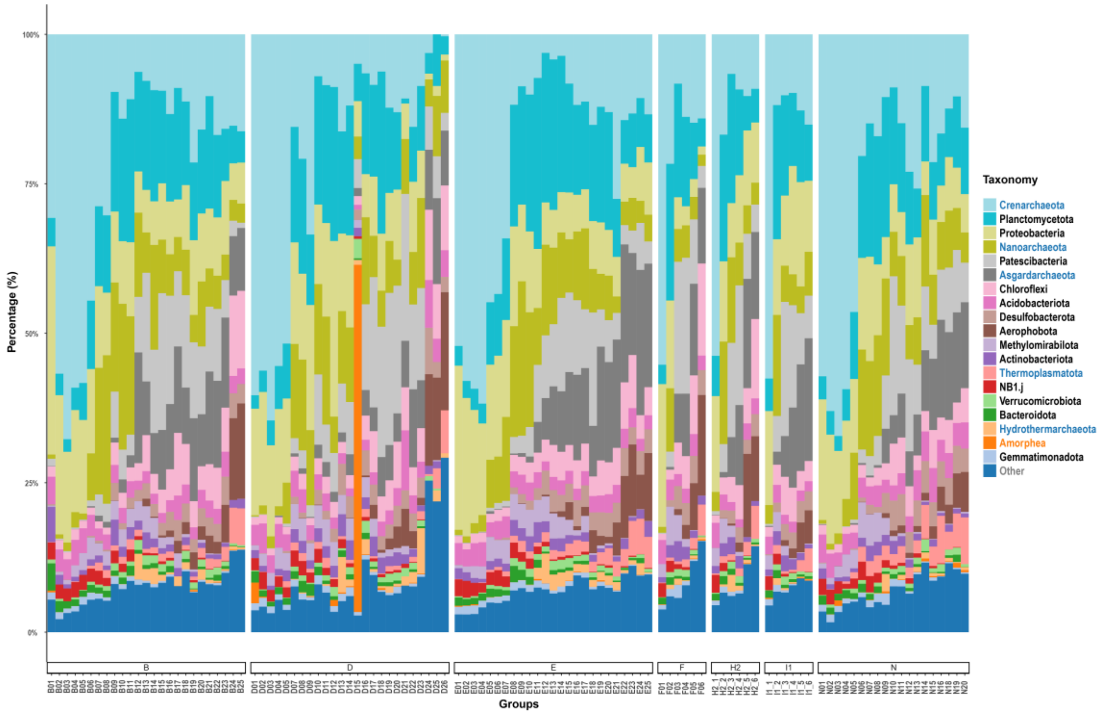

Fig. S2 Bar plot shows the 19 most abundant phyla in each push core and layer. The font color of the Phyla on the right represents the three kingdoms: archaea were colored blue, bacteria were colored black, and eukaryote were colored orange. Samples are grouped by the push core from which they come, and samples within the group are ordered from left to right according to their depth. The Crenarchaeota here consists mainly of mesophilic Crenarchaeota (now called Thaumarchaeota, but still called Crenarchaeota in the SILVA v138.1 database).

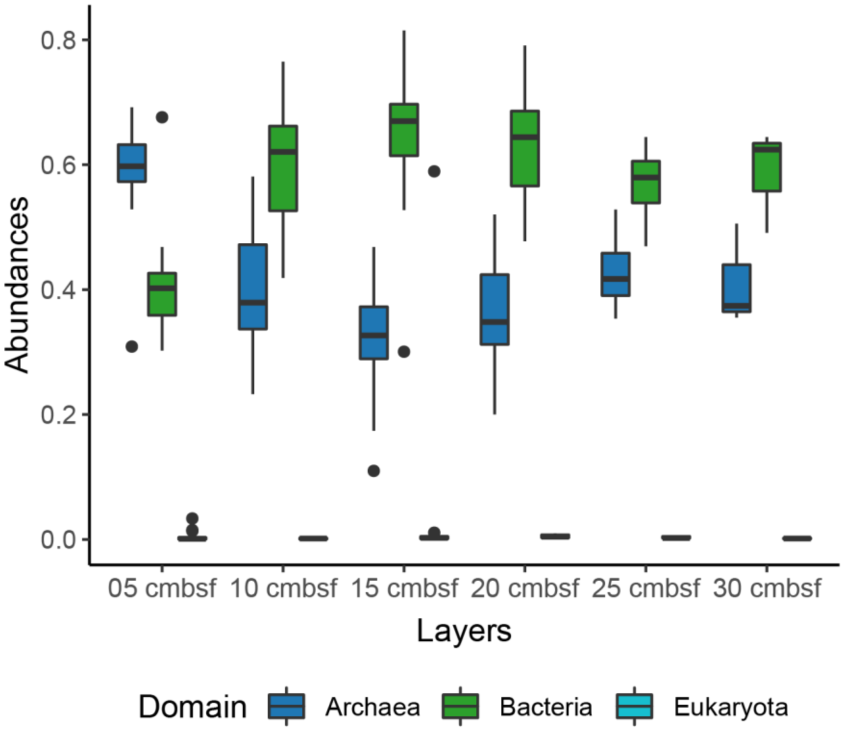

Fig. S3 Boxplot shows abundant variations of archaea, bacteria, and eukaryota between sediment layers.

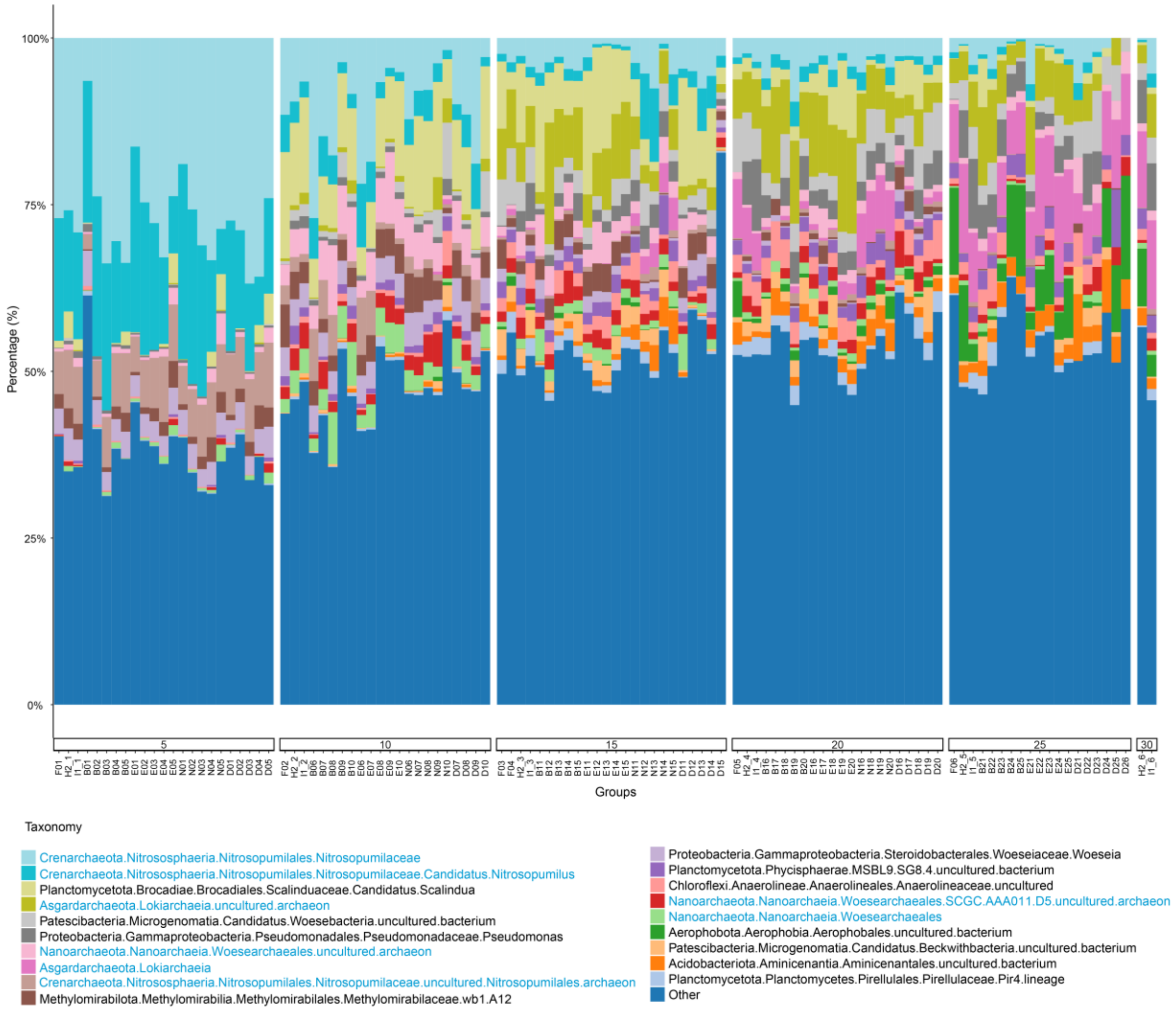

Fig. S4 Relative abundances of sequences at the genus level. The font color of the name on the bottom represents the two kingdoms: archaea were colored blue and bacteria were colored black. Samples were grouped by sediment depth (cmbsf). The Crenarchaeota here consists mainly of mesophilic Crenarchaeota (now called Thaumarchaeota, but still called Crenarchaeota in the SILVA v138.1 database).

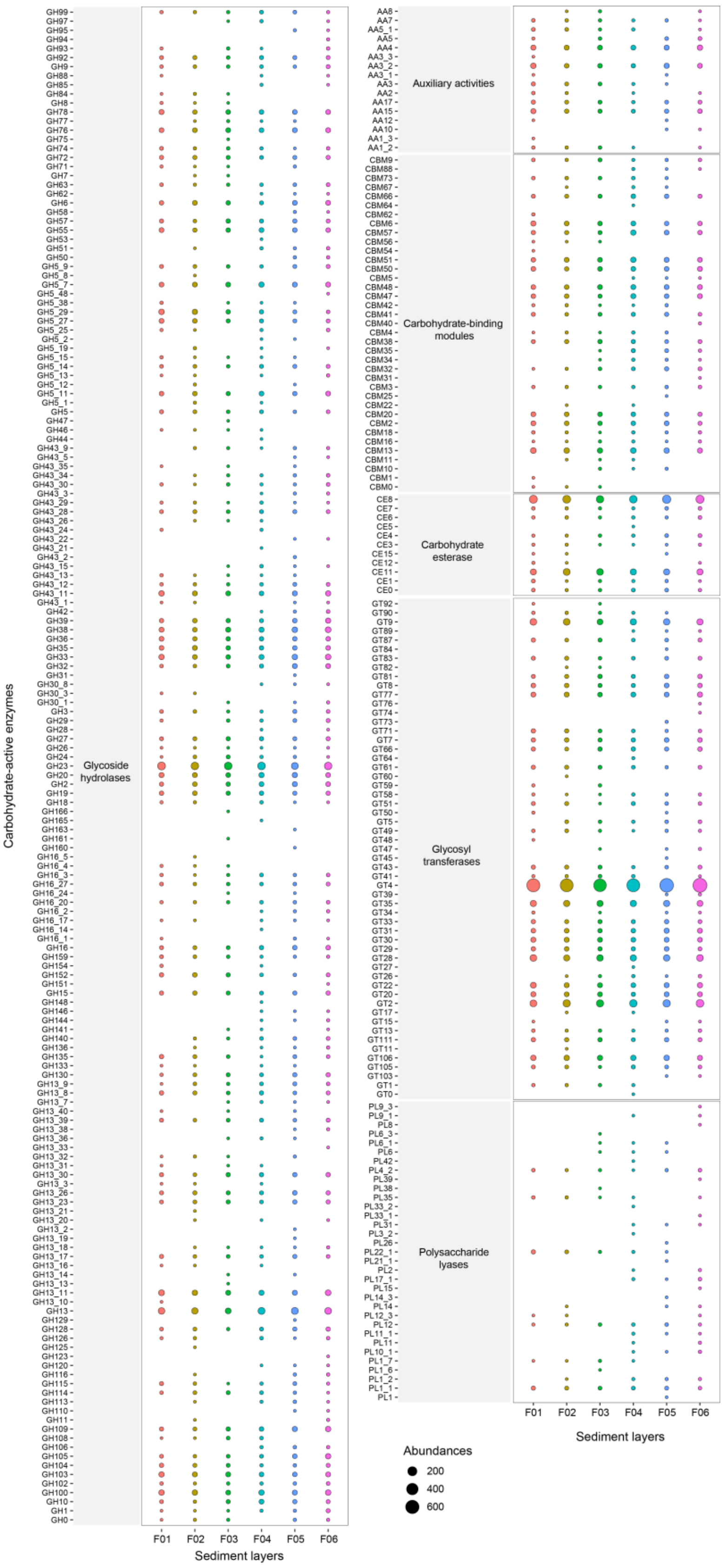

Fig. S5 carbohydrate-active enzymes in sediment layers of core F predicted by metagenomic sequencing. The enzymes were assigned to the corresponding categories according to the Carbohydrate-Active Enzymes (CAZy) database.

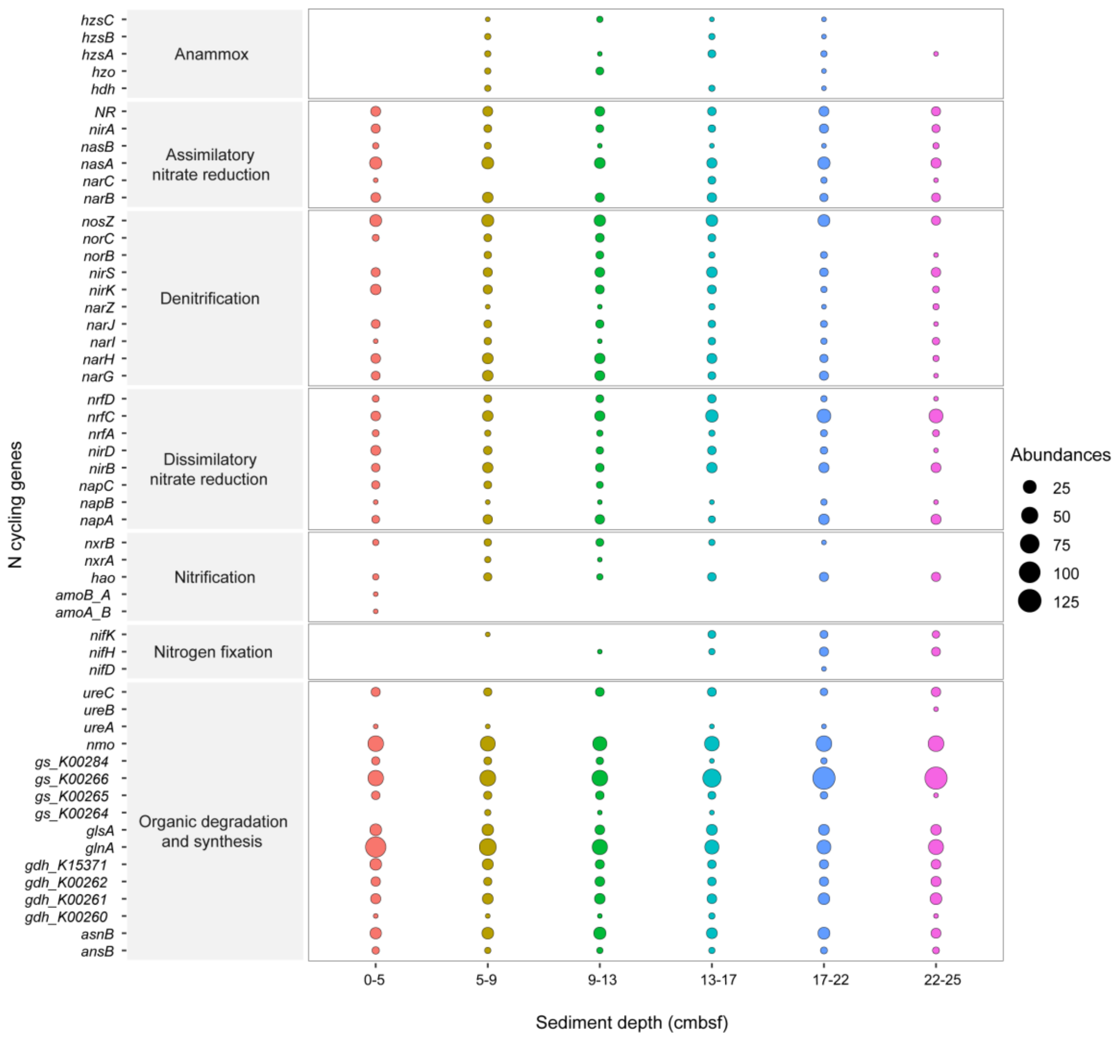

Fig. S6 nitrogen cycling genes in sediment layers of core F predicted by metagenomic sequencing. The total number of sequences was normalized to 42,996 per sample for analysis.
